# Poly-Arginine Tails and Helical Segments of Natural Antimicrobial Peptides Display Concerted Action at Membranes for Enhanced Antimicrobial Effects

**DOI:** 10.1021/acsbiomedchemau.5c00084

**Published:** 2025-07-08

**Authors:** Navleen Kaur, Kinjal Mondal, Megan E. Mitchell, Sarala Padi, Jeffery B. Klauda, Antonio Cardone, Frank Heinrich, Christina R. Harris, David K. Giles, Mary T. Rooney, Erik B. Watkins, Myriam L. Cotten, David P. Hoogerheide, Mihaela Mihailescu

**Affiliations:** † 145780 Institute for Bioscience and Biotechnology Research, Rockville, Maryland 20850, United States; ‡ Institute for Physical Science and Technology, Biophysics Program, 1068 University of Maryland, College Park, Maryland 20742, United States; § Center for Neutron Research, 10833 National Institute of Standards and Technology, Gaithersburg, Maryland 20899, United States; ∥ Information Technology Laboratory, 10833 National Institute of Standards and Technology, Gaithersburg, Maryland 20899, United States; ⊥ Department of Chemical and Biomolecular Engineering, 1068 University of Maryland, College Park, Maryland 20742, United States; # Department of Physics, 6612 Carnegie Mellon University, Pittsburgh, Pennsylvania 15213, United States; ∇ Department of Biology, Geology, and Environmental Science, The University of Tennessee at Chattanooga, Chattanooga, Tennessee 37403, United States; ○ Department of Applied Science, William & Mary, Williamsburg, Virginia 23185, United States; ◆ Oak Ridge National Laboratory, Oak Ridge, Tennessee 37830, United States; ¶ Department of Biochemistry and Biophysics, Oregon State University, Corvallis, Oregon 97331, United States

**Keywords:** antimicrobial peptide, poly arginine, AMP database, MIC, lipid bilayer, X-ray, neutron reflectometry

## Abstract

Sequence motifs or patterns found in natural antimicrobial peptides (AMPs) have a great impact on their microbicidal activities. Here, through database inquiries and biological assays, we explore the enhanced antibacterial function associated with poly arginine (poly-R) motifs that typically occur as 3–5 concatenated R residues in many natural AMPs. Using a suite of biophysical techniques, we explore the structural consequences of a C-terminal poly-R motif at membranes and correlate our findings with the functional assays. We use natural peptides, such as Tilapia piscidin 4 (TP4), as an example of how various segments in an AMP play separate and synergistic roles to achieve unmatched bactericidal effects. The function of the poly-R segment is highly consequential since the simple addition of a five-arginine (R5) tail to an otherwise inert and weakly binding helical peptide creates a potent AMP. We investigate interactions of AMPs with lipid bilayers mimicking bacterial membrane compositions, including lipopolysaccharides, to show that the poly-R tail has a key role in initiating membrane destabilization through lipid segregation and water sequestration effects, all of which facilitate insertion and translocation of the amphipathic, α-helical N-terminal segment through the membrane. We compiled a large set of natural AMP sequences and MIC values to show that, statistically, the poly-R sequence motifs have, in average, a greater impact on the overall antimicrobial efficacy than equivalent sequences with poly-K motifs and similar charge densities. We discuss our observations in light of the unique structural and hydration properties of arginine residues.

## Graphical abstract

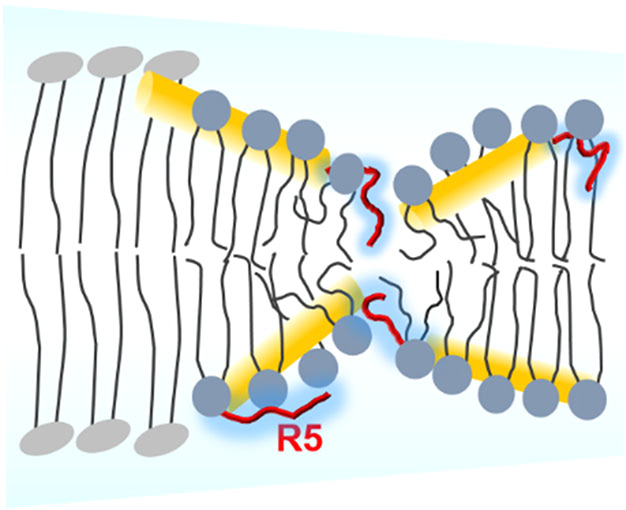

## Introduction

Microbial resistance, first identified as early as 1940,^1^ soon after the discovery of penicillin, has become a major threat to public health,^2,3^ while the discovery of new classes of antibiotics has slowed down since the 1980s.^4,5^ Among viable avenues for discovering new antimicrobials and anti-infective strategies, treatments based on antimicrobial peptides (AMP) are becoming increasingly attractive alternatives to conventional antibiotics.^6,7^ Of particular interest are cationic, membrane-active AMPs that can evade microbial resistance due to their membrane-disruptive, nonreceptor-specific mechanisms of action (see reviews^8,9^ and the references therein).

Natural AMPs, also known as host-defense peptides (HDP), are key components of all living organisms’ innate immunity. HDPs offer starting models of the structure–function relationships of AMPs at microbial lipid membranes. Drawing general principles from the relationships between sequence, structure, and activity is paramount for developing accurate prediction (computational) tools for rationally designing AMP-based antibiotics.

The availability of tens of thousands of natural and synthetic sequences with antimicrobial properties, coupled with microbial susceptibility test values (e.g., minimum inhibitory concentrations, MIC), allowed us to identify sequence motifs that stand out as important in improving antimicrobial profiles (see Methods and Results). We note that short stretches of concatenated R (poly-R sequence motifs), usually 3–5 residues long, or otherwise, densely clustered R residues, sometimes in combination with K or H, are present in some of the most potent AMPs from a variety of organisms, including marine animals,^10–12^ insects^13,14^ and humans.^15,16^ Interestingly, the poly-R motif has been exploited as a modification to vancomycin (a last resort peptide-based antibiotic in clinical use) for improved properties against persister cells and biofilms and reduced incidence of vancomycin resistance.^17,18^ Overall, the poly-R motif appears to sharpen the antibacterial activity profile with little or no toxicity in animal models, a highly desirable property for clinical use.^10,19,20^

Mature antimicrobial peptide sequences that contain poly-R motifs have been found either by direct extraction and purification of the peptides from the various organisms^10,11,13,21^ or genome analysis, cloning and expression.^12,19^ Among those, piscidins, which are fish AMPs, combine into a rich family of sequences with a high degree of homology but widely varying antimicrobial activities and efficacies. Hence, they provide some of the best examples of structure–function relationships to be used for building and validating AMP prediction models. Several members of the piscidin family share poly-R, or poly-R/K/H motif at the C-terminal end. Among these members are some of the most potent AMPs: Chrysophsins, isolated from the gills of red sea bream;^11^ Misgurin,^10^ isolated from mudfish; piscidins such as P1, isolated from the mast cells of hybrid striped bass;^22^ and a close relative, Nile Tilapia piscidin 4 (TP4), found by isolation of cDNA clones.^19^ They often display MIC values much lower than those reported for many other AMPs, and they are usually highly active against resistance-prone bacterial strains, such as methicillin-resistant *Staphylococcus aureus* (MRSA).^23,24^ Chrysophsin-1, -2 and -3 display a C-terminal HRRRH-motif^11^ and an amidated C-terminus, a common post-translational modification found in mature, natural AMPs.^25^ Misgurin, bearing an RRRK motif in the C-terminus, shows a broad-spectrum antimicrobial activity in vitro, which was found to be about six times more potent than magainin 2.^10^ TP4, carrying an RRRRR (R5) motif at the C-terminal end, exhibits cell proliferation stimulating, wound closure-inducing, and bacterial infection-reducing activity.^20,23,24^

All evidence suggests that the poly-R motif plays an important role in host immunity during bacterial infection. However, the role of poly-R segments found in natural AMPs in interactions with bacterial membranes has been only broadly addressed under the general concept of “cationicity”. Here we are addressing the specific role of poly-R segments in interaction with membranes and in relation with the more hydrophobic segments that occur in AMP peptides. We also make the distinction between poly-R segments found in synthetic AMPs or cell penetrating peptides (CPP) with antimicrobial properties, and natural antimicrobial peptides that carry poly-R motifs and are the focus of this work. We explore the specific role of such charged segments, often found at the N- or C-termini of the most potent natural AMPs, in the mechanisms of action at bacterial membranes.

Understanding the molecular basis for AMP actions at bacterial membranes requires examination of specific interactions within the complex milieu presented by various bacterial species. Gram-positive bacteria are surrounded by a thick layer of peptidoglycan that forms a protective shell around the cytoplasmic, inner membrane (IM). The IM of different bacterial species can vary substantially in composition, but typically include large amounts of anionic lipids, such as phosphatidylglycerol (PG) and cardiolipin (CL). All these membrane layers constitute strong attractors for cationic peptides. Gram-negative bacteria have a more complex structure, with a double membrane (outer and inner) and a thin peptidoglycan layer in the periplasmic space. The outer membrane (OM) is asymmetric, with the inner monolayer made of glycerophospholipids and the outer monolayer composed mainly of lipopolysaccharide (LPS).^29^ The inner membrane is made of a mixture of anionic and zwitterionic phospholipids, such as PG and phosphatidylethanolamine (PE). While the complexity cannot be reproduced in vitro, model membranes designed in the laboratory can offer useful insights into specific interactions and events that lead to bacterial membrane destabilization and permeabilization. Biophysical and structural studies in model membranes are especially useful when matched with cell-based assays in bacterial cultures.

To couple measurement results with statistical data derived from AMP sequences containing poly-R motifs, we gathered and analyzed a large amount of sequence information and bioactivity data from several sources (Table S1). Our statistical analysis focuses on natural AMP sequences, although we also touch on synthetic AMPs. Experimentally, this study explores the structural basis for the added functionality of AMPs by poly-R segments by using TP4 and P1 (a close homologue of TP4, that naturally lack the R5 tail) peptides from teleost fish as models. We utilized peptide sequence variants with and without five consecutive Arginine (TP4, TP4-noR5, P1, P1-R5) at the C-terminal end. Furthermore, to parse out the contribution of the R5 tail alone, we examined the antimicrobial properties of a model peptide inspired by the work of Baldwin et al.,^26^ herein called neutral peptide (NP). NP is an alanine-rich peptide that adopts a stable, helical structure in solution and binds very weakly to lipid bilayers.^27,28^ We modified the NP peptide by appending an R5 motif at its C-terminus, producing “NP-R5”, to observe the changes to the antimicrobial efficacy and its interaction with model membranes. Antimicrobial assays were performed on both Gram-positive and Gram-negative bacteria to assess minimum inhibitory concentrations of the peptides. A wide range of biophysical and structural measurements were conducted on bacterial membrane mimics (liposomes and flat lipid bilayers of varying composition, including *Salmonella* rough LPS) to examine the correlation between the antimicrobial efficacies and the structural perturbations inflicted on bilayers, with emphasis on the poly-R motif contributions.

## Materials and Methods

### Materials

The peptides (TP4, TP4-noR5, NP, NP-R5) were chemically synthesized, purified to >95% purity and converted to HCl salt, by Biomatik (Wilmington, Delaware). The peptides were dialyzed to eliminate all the salts, and their concentration was determined using amino acid analysis. A NanoDrop UV–vis spectrometer was also used with absorbance recorded at 205 nm for peptides without tryptophan and at 280 nm for those containing tryptophan using the previous stocks as standard. The TP4, P1-R5, and P1 peptides used for the dye leakage assays and NMR experiments were synthesized and purified by the Peptide Chemistry Technology Center at the University of Texas Southwestern Medical Center. The labeled Gly (^15^N, 98%) was purchased from Cambridge Isotope Laboratories (Tewksbury, MA, USA). Each peptide was dissolved in dilute HCl to neutralize trifluoroacetate ions and generate the chloride salt form of the peptide. Dialysis was performed to remove excess chloride ions.^30^ Following extensive lyophilization, the peptide was dissolved in Nanopure water and sent for amino acid analysis at the Protein Chemistry Center located at Texas A&M (College Station, TX). All peptides used were carboxyamidated.

Phospholipids including 1-palmitoyl-2-oleoyl-glycero-3-phosphocholine (POPC), 1-palmitoyl-2-oleoyl-*sn*-glycero-3-(phospho-rac-(1-glycerol)) (POPG), 1,2-dipalmitoyl-*sn*-glycero-3-phosphoglycerol, sodium salt (DPPG), 1-palmitoyl-2-oleoyl-*sn*-glycero-3-phosphoethanolamine (POPE), and 1,2-dioleoyl-*sn*-glycero-3-phosphoethanolamine (DOPE) were sourced from Avanti Polar Lipids (Alabaster, AL). Lipopolysaccharide (LPS-Re) derived from *Salmonella Minnesota R595* (Ultrapure-LPS, free of lipoprotein) was purchased from InvivoGen (San Diego, CA). By Mass Spectrometry, we found a major peak corresponding to a molecular mass of 2523 g/mol. and the presence of a few other species. Bacterial strains were purchased from Microbiologics (San Diego, CA). Mueller-Hinton broth (MHB) was bought from Hardy Diagnostics (Santa Maria, CA). Bacto agar was purchased from Becton Dickinson and Company (Sparks, MD). Tryptic soy broth (TSB) and reagents like sodium hydroxide, sodium phosphate monobasic monohydrate, sodium phosphate dibasic, propidium iodide, calcein, and sodium chloride were all purchased from Sigma-Aldrich (Rockville, MD).

### Methods

#### Statistical Analysis of AMP Sequences from Databases

To analyze the effect of positively charged amino acid motifs on the antimicrobial activity of peptides, we compiled a data set of AMP sequences along with their respective MIC values. Table S1 in the Supporting Information (SI) provides a list of the databases and online sources we utilized for data collection. The data was initially sourced from three different databases: (i) GRAMPA,^31^ which includes information from other databases such as the Antimicrobial Peptide Database (APD),^32^ Database of Antimicrobial Activity and Structure of Peptides (DBAASP),^33^ YADAMP,^33^ and DRAMP;^33^ (ii) StarPep;^34,35^ and (iii) DBAASP3.^36^ We identified all naturally occurring peptide sequences along with their MIC values using the ‘Ribosomal’ Keyword in DBAASP. All MIC data were standardized to μmol/L. Table S2 in the SI presents the statistical data on both naturally occurring and synthetic peptide sequences. In total, we curated 7812 sequences, of which 2486 are natural, and 5326 are synthetic sequences. Data processing and statistical analysis was done using Python programming language.

#### Minimum Inhibitory Concentrations of Peptides

Minimum inhibitory concentrations were determined by broth microdilution technique.^37^ Two bacterial species were used: Gram-positive bacteria *Staphylococcus epidermidis* (*S. epidermidis*), derived from ATCC 14990 and Gram-negative bacteria *Escherichia coli* (*E. coli*) MG1655, respectively. *S. epidermidis* was grown in TSB, and *E. coli* was grown in MHB. Both were stored at 4 °C on their respective growth medium agar plates. Before the assay, bacteria were grown overnight in 5 mL of growth medium at 37 °C. The grown bacteria were further subcultured in 3 mL of growth medium by diluting to 1% until the O.D. reached 0.4–0.6 within 2.5–3 h. After the bacteria reached the mid log phase, further dilution was made to attain an O.D. of 0.001 to get a final of ∼10^5^ CFU/mL in each well of a 96-well plate.^38^ An Opentron OT_2 robot pipetting system was used to automatically prepare serial dilutions of peptides and add the bacteria to attain a total volume of 100 μL in each well. The wells containing only medium served as a negative blank. The plate was then incubated for 24 h at 37 °C, and the O.D. of the plate was recorded at 600 nm using a BioTek (Agilent, Santa Clara, CA) microplate reader (Figure S1). The lowest concentration at which no bacterial growth was observed was considered the MIC of the peptide.

#### Antimicrobial Peptide Susceptibility Assay on *Vibrio cholerae*

*Vibrio cholerae* (*V. cholerae*) O1 El Tor strain C6706 cultures were grown in CM9-Hepes (pH 7.4) minimal media (100 mmol/L Hepes, 0.4% glucose, 0.4% casamino acids) to OD_600_ of approximately 0.8–0.9. Cultures were centrifuged, washed with media, and used to create the inoculum for the assay. Dilutions of each antimicrobial peptide were prepared in LoBind tubes in CM9-Hepes. Polypropylene microtiter plates were prepared by adding 30 μL of either media or one of the antimicrobial peptide dilutions followed by 170 μL of prepared cultures to wells at the standard inoculum (5 × 10^5^ cfu/mL) for microtiter broth dilution MIC determination.^39^ Plates were incubated shaking at 37 °C for 20 h. Cultures were transferred to polystyrene microtiter plates before reading on a BioTek Synergy microplate reader at 600 nm. Experiments were performed in triplicate and repeated with three biological replicates. Bacterial viability and death were further verified by sampling individual wells at each antimicrobial concentration for growth on Luria agar.

#### Propidium Iodide Uptake by Bacterial Cells

Propidium iodide (PI) dye was used in this assay to test the permeabilization of bacterial membranes or bactericidal activity. The dye was mixed with the bacterial cells (∼10^8^ cells/well) in a 96-well plate. The final concentration of propidium iodide was 4 μmol/L in each well, along with the peptides at 5 μmol/L in 10 mmol/L sodium phosphate buffer, pH 7. For this assay, the bacteria were grown overnight, and then a secondary culture was grown to reach an optical O.D. of ∼0.6, as described above. The obtained bacterial culture was centrifuged for 10 min at 4000 rpm and 4 °C and then resuspended in buffer (10 mmol/L sodium phosphate buffer, pH 7). The process was repeated to remove any residual growth medium, and the final O.D. was determined to be close to 0.5. The fluorescence intensity of propidium iodide was measured at 617 nm when excited at 535 nm using a BioTek plate reader. Control (blank) measurements of bacteria alone and buffer alone (without dye) were also done. The fluorescence intensities were normalized with that of the intensity obtained for buffer alone.

#### Dye Leakage Assay

Calcein leakage assays were done on large unilamellar vesicles (LUVs) following a procedure previously described.^40,41^ Briefly, a 3:1 POPC/POPG lipid film (containing 4 μmol total of lipids) was prepared in a round-bottom flask using the lipids dissolved in chloroform. The solvent was removed under N_2_ gas before drying overnight under vacuum. The next day, the lipid cake was hydrated with 300 μL of 80 mmol/L calcein dye (pH 7.4) to generate a suspension that was subjected to 3 freeze–thaw cycles before extrusion through a 0.1 μm polycarbonate filter placed in a mini extruder (Avanti Polar Lipids). Untrapped calcein was separated from the LUVs by chromatography using a Sephadex G-50 column run with HEPES buffer (50 mmol/L, 100 mmol/L NaCl, 0.3 mmol/L EDTA, pH 7.4). A total phosphorus assay was performed to determine the exact lipid concentration of the LUVs.^42^ After diluting the vesicles to a final concentration of ∼35 μmol/L, the LUVs were plated in 180 μL wells and exposed to 20 μL of TP4 solutions serially diluted from stock, as needed to generate peptide-to-lipid ratios (P/L) ranging from P/L = 1:2 and 1:1024. Triplicates of each well were run, and at least three independent assays were performed as well. The 96-well plate was incubated at 20 °C with shaking for 40 min. After equilibration at room temperature, the fluorescence was measured using a BioTek SynergyH1 plate reader (BioTek, Winooski, VT) using excitation and emission wavelengths of 490 and 520 nm, respectively. The fractional leakage produced by TP4 was calculated using the equation:

$$\%\mathrm{leakage}=\frac{I_{x}-I_{0\%}}{I_{100\%}-I_{0\%}}$$

where *I*_*x*_ is the fluorescence intensity in a peptide-containing well, *I*_100%_ is the intensity of the positive control (100% leakage obtained with 20 μL of 1% Triton-X detergent), and *I*_0%_ is the intensity of the negative control (0% leakage obtained with 20 μL nanopure water). P1 was run in parallel for comparative purposes. The dose–response curves were fitted in GraphPad Prism using a modified version of the Hill equation to extract the EC_50_ as the P/L where half of the dye has been released.^43^ GraphPad Prism also provided the 95% confidence interval (CI) for the EC_50_ values.

#### Lipid Sample Preparations

##### Preparation of Lipid Vesicles

Lipids were codissolved in chloroform or mixtures of methanol and chloroform at the desired molar ratio. The solvent was removed under a gentle stream of nitrogen gas, followed by residual solvent removal under a vacuum to obtain a dry lipid film. The dried lipids were then hydrated with water or 10 mmol/L sodium phosphate buffer, pH 7 and vortexed repeatedly to form multilamellar vesicle suspensions of desired concentrations. Small unilamellar vesicles (SUVs) were obtained by sonication of the vesicle suspension for 15–20 min using a bath sonicator and gentle heating at a temperature above the gel-to-fluid phase transition of the lipid, when necessary, until a clear solution was obtained. LUVs were obtained by extruding the vesicle suspension 11–21 times through a polycarbonate membrane of 100 nm pore size using a mini-extruder (Avanti Polar Lipids).

##### Preparation of Samples for X-ray Diffraction

SUV suspensions were prepared as above, creating stocks of lipids in pure water, which could be further diluted with the buffer of interest. Peptide stocks in water were produced by solubilizing a small amount of peptide powder in pure, doubly distilled water, followed by dialysis against pure water and concentration determination by amino acid analysis. Samples of higher concentration than the initial stock were prepared by lyophilizing desired amounts of peptide and resuspending in water/buffer at the desired new concentration. Absorbance at 205 nm was employed, with extinction coefficients calculated according to,^44^ to verify the new concentration using the initial stock as a standard for calibration. Peptide was added to a concentrated solution of lipid vesicles (5–10 mg/mL) in the same buffer at the desired P/L and allowed to incubate before spreading the mixture on thin glass coverslips. The bulk water was allowed to slowly evaporate by allowing the samples to sit overnight at room temperature. This procedure creates oriented bilayer stacks (lamellar samples), typically made of 1000–2000 bilayers, with peptides populating both sides of the bilayers (Figure 1) through vesicle fusion, transient pore formations and exchange, and molecular exchanges. The same stock solution of lipid was used as blank (neat lipid, without peptide). Before performing the diffraction experiments, the samples were placed in an enclosed chamber and annealed at 93% relative humidity and room temperature for several hours.

**Figure 1:**
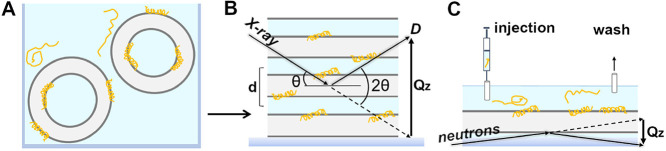
Illustration of sample preparation platforms used for measurements in model lipid membranes. (A) Sample used for spectroscopic and thermodynamic measurements (CD, fluorescence, and DSC) were in the form of lipid vesicles (SUVs or LUVs). (B) Oriented multilayers (lamellar samples) were produced from lipid vesicles incubated with peptides, as shown in (A), see Methods. Diffraction data were taken by varying the incidence angle (θ) and the detector angle (*D*) at the same time, thus probing the structure on the *z*-axis, normal to the bilayer plane. *Q*_z_ is the momentum transfer for the diffracted photons, *d* is the repeat spacing of the multilayered system (see below). (C) Supported single bilayer system used for neutron reflectometry measurements.

##### Preparation of Samples for Neutron Reflectometry

Lipids in powder form were stored at −80 °C prior to use. Stocks were made immediately prior to the experiment (10 mg/mL DOPE in isopropanol and 1 mg/mL POPG in ethanol) and mixed to 75% DOPE, 25% POPG at a total lipid concentration of 1 mg/mL in isopropanol. The final concentration of ethanol from the POPG was 25%. Buffers used were 10 mmol/L Tris pH 7.4 in either H_2_O or deuterium oxide (heavy water, D_2_O), and 10 mmol/L tris pH 7.4 with 150 mmol/L sodium chloride in either H_2_O or D_2_O. Peptide was dissolved in the tris buffers (without sodium chloride) to a concentration of 3 μmol/L. Bilayers were formed by the solvent assisted bilayer formation method (SALB).^45–47^ Flow cells were filled with 1 mg/mL 3PE:1PG lipid mixture in 75% isopropanol, 25% ethanol. With the flow cell maintained in a vertical orientation to ensure complete fluid exchange,^48^ 10 mmol/L tris (pH 7.4) with 150 mmol/L sodium chloride in D_2_O was flown in from the bottom of the cell at a rate of 0.04 mL/min. After bilayer formation (Figure 1C), subsequent buffer exchanges were performed manually or via syringe pump at 2 mL/min. For peptide additions, at least 4 mL of 3 μmol/L peptide was injected (to avoid depletion of the peptide solution) over the course of 10 min.

#### DSC Measurements

SUVs prepared as above from one single lipid or mixture of lipids were sonicated using a bath sonicator for 15–20 min at temperatures above the gel-to-fluid transition temperature of all the components. The vesicle suspensions were allowed to further hydrate overnight in the refrigerator. The samples were measured at a lipid concentration in the range of 1–2.5 mg/mL. DSC measurements were made on VP-DSC microcalorimeter (MicroCal Inc., Northampton, MA). For samples containing peptides, the peptide was added to the lipid vesicles suspension in water at room temperature, allowed to equilibrate on the bench with gentle mixing, followed by degassing and precooling to the desired starting temperature of the first DSC scan. At least four consecutive scans, two heating and two cooling, were taken for each sample, at a scan rate of 30 °C/h. There was a delay of 5 min between sequential scans to allow for thermal equilibration. DSC curves were analyzed (corrected for baseline and normalized to concentration) by Origin, version 7.0 (OriginLab Corporation).

#### Circular Dichroism Spectroscopy

CD spectroscopy was employed to study the secondary structure of peptides in the absence and presence of lipid vesicles (Figure 1A). CD spectra of TP4 and TP4-noR5 were recorded in the presence of LPS-Re and LUVs of POPC/LPS-Re at a molar ratio of 5:1, a peptide concentration of 10 μmol/L and a P/L molar ratio of 1:30 in both the cases. The quality of the spectra and the subsequent analysis is impaired by light scattering effects, therefore, the lipid and peptide concentrations were kept to a minimum and only the region between 190 and 260 nm were analyzed. The CD spectra were recorded over a wavelength range of 190–260 nm, with a spectral bandwidth of 1 nm and the time per point set at 5 s. All the measurements were done at 25 °C. The background contributions (lipid in buffer) were subtracted from sample spectra (with peptide). CD data, measured in units of millidegrees, were converted into molar ellipticity per residue (MRE) to calculate fractional helical content: *f*α = (MRE – MRE_RC_)/(MRE_H_ – MRE_RC_), where MRE_RC_ and MRE_H_ are the limiting values for a completely random coil and a completely α-helical conformations at 222 nm. Here, the following values were considered: MRE_RC_ = −1500 deg/cm^–^^2^ dmol and MRE_H_ = −33,400 deg/cm^–^^2^ dmol.^49^

#### Solid-State NMR

##### Preparation

Oriented samples were prepared as described previously.^50–52^ Briefly, 3:1 POPC/POPG lipid films (∼20 mg lipid total) were prepared using stocks of the lipids dissolved in chloroform. The organic solvent was removed using N_2_ gas, and the samples were placed under vacuum overnight. Each film was then hydrated with 10 mL of Bis-tris buffer (3 mmol/L, pH 7.4), and the resulting suspension was incubated overnight at 40 °C. The next day, the suspension was centrifuged at 8 °C for 1.5 h using a Beckman Optima-90K centrifuge and a Beckman SW40Ti rotor set at 23,700 rpm. After removing the supernatant, the pellet was spread on 15–20 thin glass slides (dimensions 5.7 × 12 × 0.03 mm^3^ from Matsunami Trading Co., Japan). The sample was equilibrated in a chamber maintained at a relative humidity >90% using a saturated solution of potassium sulfate. Each slide was rehydrated with the supernatant to achieve 40% hydration by weight. Following stacking, the slides were inserted in a glass cell (6 × 20 × 4 mm^3^, Vitrocom Inc., NJ) before sealing with beeswax (Hampton Research, Aliso Viejo, CA). The sample was incubated at 40 °C until it appeared homogeneously hydrated.

##### Experiments

2D heteronuclear correlation (HETCOR) spectra were obtained on a 750 MHz wide bore Bruker instrument with Avance 1 console at William & Mary. The data were collected at 32 ± 0.1 °C using parameters typically used on piscidin samples,^53^ including a recycle delay of 5 s and 32–48 *t*_1_ increments, and 896 transients each. A ^1^H radiofrequency amplitude of 83.0 kHz was used during the MSHOT ^1^H homonuclear decoupling^54^ in the *t*_1_ (^1^H) dimension and SPINAL decoupling in the *t*_2_ (^15^N) dimension. By setting the delay τ_d_ to 6.3 μs, a dwell time of 42.6 μs was used in the indirect dimension. To enhance the detection of the ^15^N/^1^H dipolar coupling, the ^1^H carrier frequency was centered on the amide proton region of the peptide (i.e., ∼15 ppm). Magnetization transfer from the amide proton to its closest ^15^N spin was accomplished using a WIM-12 (*w*indowless isotropic mixing) sequence featuring a ^1^H and ^15^N radiofrequency amplitude of 55 kHz and a short mixing time (∼100 μs). Processing was done using 50 Hz of Gaussian line-broadening in the ^15^N dimension. The spectra were referenced to a saturated aqueous solution of (^15^NH_4_)_2_SO_4_ set at 26.8 ppm with respect to liquid NH_3_.

#### X-ray Diffraction Measurements

XRD measurements were carried out with a 3KW Rigaku Smartlab diffractometer, located at IBBR, Rockville, Maryland. Lamellar samples were produced as described above (Figure 1B). Diffracted intensities were recorded as a function of angle by maintaining the incidence angle and the detection angle equal to each other (θ–2θ mode, Figure 1B), which probes the bilayer structure projected along the normal to the bilayer plane (*z*-axis). Repeat spacings (*d*) and their uncertainties were determined by a linear fit of the Bragg peak position versus diffraction order for the most prominent peaks. Bragg peaks are observed at angles where momentum transfer *Q*_z_ = 4πλ^–1^sin­(θ) is equal to *h*(2π/*d*), where *h* is the diffraction index, and λ is the photon wavelength, here 1.54 Å. Structure factors were calculated from the integrated Bragg intensities after removing the background and applying Lorentz, polarization, beam footprint, and absorption corrections for all observable peaks. Phases of the structure factors were determined using the swelling method^55,56^ (see Figure S4A–C for an example). The one-dimensional electron density profiles of the hydrated bilayers along the *z*-axis were calculated on an arbitrary scale using direct Fourier reconstruction.^57^

#### Neutron Reflectometry Measurements

Neutron reflectometry experiments were performed on the LIQREF horizontal reflectometer at the Spallation Neutron Source at Oak Ridge National Laboratory. Liquid flow cells consisted of a silicon backing wafer and sample wafer (each 5 mm thick and 2 in. in diameter) separated by a nominally thin Viton gasket, forming a reservoir with an estimated volume of 0.22 mL. Holes were drilled in the backing wafer for the inlet and outlet. Sample wafers were single-crystal silicon with a native oxide surface.

A polychromatic beam of neutrons impinged on the interface between the surface of the sample wafer and the liquid in the sample cell reservoir. Each measurement covered a range in scattering wavevector *Q* = 4πλ^–1^sin­(θ) from 0.010 to 0.287 Å^–1^, binned to maintain a constant bin width equal to 1.0% of the bin center.

Four measurements were performed on each bilayer, one in each D_2_O and H_2_O before the peptide was added, and the same two contrasts thereafter. NR data were analyzed using composition space modeling described previously.^58^ Briefly, the composition space model arranges the known molecular components of the bilayer and protein at the substrate surface; any unfilled space is filled with water. Because the neutron scattering length density (nSLD) of each component is known from its elemental composition and molecular volume, an average nSLD profile can be calculated as a function of distance from the substrate surface. This nSLD profile, in turn, corresponds to a predicted R­(Q), and can be optimized to fit the experimental data, using as parameters the spatial arrangement of the molecular components. Replacing all H_2_O in the membrane-bathing buffer with D_2_O provides scattering contrast and allows for an unambiguous determination of the nSLD profile.^59^ Here, all four conditions (H_2_O and D_2_O, before and after peptide addition) were simultaneously analyzed, with additional parameters describing the spatial distribution of the peptide and its structural effect on the bilayer. Model construction, statistical analysis, and image generation were performed using the *molgroups* (https://github.com/reflectometry/molgroups) plugin to *Refl1D* software (https://github.com/reflectometry/refl1d). Model optimization was performed using the DREAM Markov Chain Monte Carlo algorithm.^60^ Confidence intervals (CI) on parameters and model predictions were calculated from parameter distributions derived from 5000 DREAM samples after the optimizer had reached a steady state.

## Results

### Statistical Analysis of Peptide Sequences

We conducted a statistical analysis for naturally occurring AMP sequences in order to find relationships between the amount of positive charge carried by AMPs and their antimicrobial efficacies, with an emphasis on clustered charge motif effects. We have collected MIC data for 2486 natural and 5326 synthetic sequences with reported MIC values for various types of bacteria (Table S2), as discussed in Methods. For each AMP in our collection, we calculated an average MIC, by taking the mean of all available MIC values for that particular AMP and various bacteria species (Bacteria Averaged). Furthermore, in order to match the analysis with our experimental data, we separated MIC values for specific targets (*E. coli* and *S. epidermidis*). We focused on three separate segments of each peptide sequence: the N-terminal third (START), the second third (MIDDLE), and the C-terminal third (END). For each segment, we identified all sequences containing either poly-R or poly-K motifs of a given size (*n*) and we computed mean and standard error of the associated MIC values accordingly. We included sequences with *n* = 1 in the analysis, for comparison and considering the higher availability of catalogued sequences in that category. Tables 1–3 summarize the results of the analysis for the MIC values, along with their standard error and p-values, as a function of poly-R or poly-K motif length, for Bacteria Averaged, *E. coli* and *S. epidermidis*, respectively. The p-value is the output of the *t* test for the null hypothesis that the mean value associated with poly-K motifs is less than that of poly-R motifs. A *p*-value less than 0.05 indicates that the mean MIC values associated with poly-R motifs are statistically lower than the ones associated with poly-K motifs (p-values equal to 1 are not reported in the tables for brevity). For instance, as far as the END segments are concerned, the MIC values associated with poly-R motifs are statistically lower than poly-K motifs of sizes 1 and 2, for both bacteria averaged and *E. coli*, but not the case for *S. epidermidis*. A similar trend is seen for the MIDDLE segments, but not for the START segment. In contrast, no clear trends emerge for any of the segments in the case of synthetic peptides (Tables S3–S5 and Table 2).

**Table 1:** MIC Values in μmol/L as a Function of poly-R/poly-K Motif Size for Natural Peptide Sequences of Bacteria Averaged^a^

	position of (*n*) consecutive arginine/lysine amino acids (bacteria averaged)					
	START		MIDDLE		END	
motif size (*n*)	R	K	R	K	R	K
R/K	18.86 ± 0.61 (210)*	21.94 ± 0.99 (536)*	17.43 ± 1.34 (203)*	24.34 ± 0.84 (755)*	17.71 ± 1.12 (321)*	25.43 ± 0.84 (832)*
RR/KK	18.53 ± 3.47 (21)	16.85 ± 3.36 (42)	15.30 ± 4.38 (26)*	28.85 ± 2.50 (100)*	11.33 ± 2.41 (36)*	21.54 ± 1.57 (171)*
RRR/KKK	13.74 ± 12.4 (2)	5.00 ± 2.29 (3)	7.68 ± 3.30 (3)	n.a.	6.33 ± 2.49 (7)**	9.60 ± 3.89 (5)**

a The mean and standard error of the mean (the standard deviation σ, divided by the square root of the number sequences containing the specified motif as listed in parentheses). ‘n.a.’ indicates that there are no sequences found with the specified motif size. We list *p*-values reporting the probability that the mean of the MIC is larger for R than K. For cases when the T-statistics return a negative value, we do not report the *p*-value. R/K: Reporting MIC values by selecting sequences with R motifs and K motifs individually. Similarly, we report MIC values for RR/KK and RRR/KKK. Note: * Indicates *p*-value is less than 0.05 and ** indicates *p*-value greater than 0.05.

**Table 2:** MIC Values in μmol/L as a Function of poly-R/poly-K Motif Size for Natural Peptide Sequences for *E. coli* ^a^

	position of (*n*) consecutive arginine/lysine amino acids (*E. coli*)					
	START		MIDDLE		END	
motif size (*n*)	R	K	R	K	R	K
R/K	16.31 ± 1.68 (157)**	18.73 ± 1.08 (460)**	16.62 ± 1.63 (149)*	21.73 ± 0.97 (623)*	16.52 ± 1.40 (245)*	23.33 ± 1.01 (643)*
RR/KK	15.33 ± 4.73 (16)	10.13 ± 3.35 (35)	15.55 ± 4.98 (17)*	28.31 ± 2.83 (92)*	7.88 ± 2.85 (25)*	23.06 ± 2.07 (132)*
RRR/KKK	n.a.	1.00 ± 0.00 (2)	10.48 ± 4.68 (3)	n.a.	1.86 ± 0.77 (6)**	5.28 ± 2.28 (6)**

a For more details, see Table 1 footnote.

**Table 3:** MIC Values in μmol/L as a Function of poly-R/poly-K Motif Size for Natural Peptide Sequences for *S. epidermidis* ^a^

	position of (*n*) consecutive arginine/lysine amino acids (*S. epidermidis*)					
	START		MIDDLE		END	
motif size (*n*)	R	K	R	K	R	K
R/K	14.59 ± 4.53 (24)**	17.17 ± 3.00 (57)**	7.36 ± 1.76 (22)*	22.16 ± 2.61 (93)*	10.92 ± 3.50 (29)**	18.06 ± 2.57 (86)**
RR/KK	7.13 ± 0.83 (2)	3.70 ± 1.10 (4)	9.75 ± 6.38 (4)**	21.01 ± 3.73 (27)**	3.92 ± 0.85 (4)**	5.71 ± 1.67 (11)**

a For more details, see Table 1 footnote.

To understand the influence of charge density on the average MIC values, as well as the difference in effects between poly-R and poly-K, we mapped the distribution of naturally occurring AMP sequences, on the two-dimensional space (ρ_+_/MIC) where the two axes were binned into reasonably small ranges. In this case, only the Bacteria Averaged was considered, for generality and because a lot more sequences were available in that category. We first identified the subset of sequences containing at least a poly-R or poly-K motif of size >1. Then, for each sequence we computed the charge density as follows: ρ_+_ = *N*_R|K_/*N*_AA_, where ρ_+_ is the charge density, *N*_R|K_ is the number of R or K residues in a sequence, and *N*_AA_ is the total number of residues. For each charge density bin, the distribution of poly-R (poly-K) sequences were normalized to their corresponding total. Subsequently, for each ρ_+_ and MIC value range, we computed the difference in relative frequency between sequences with poly-K versus poly-R. A negative value for the difference means there are more poly-R sequences than poly-Ks populating that space. As shown in Figure 2, the space with lower MIC values is predominantly populated by sequences containing poly-R motifs.

**Figure 2:**
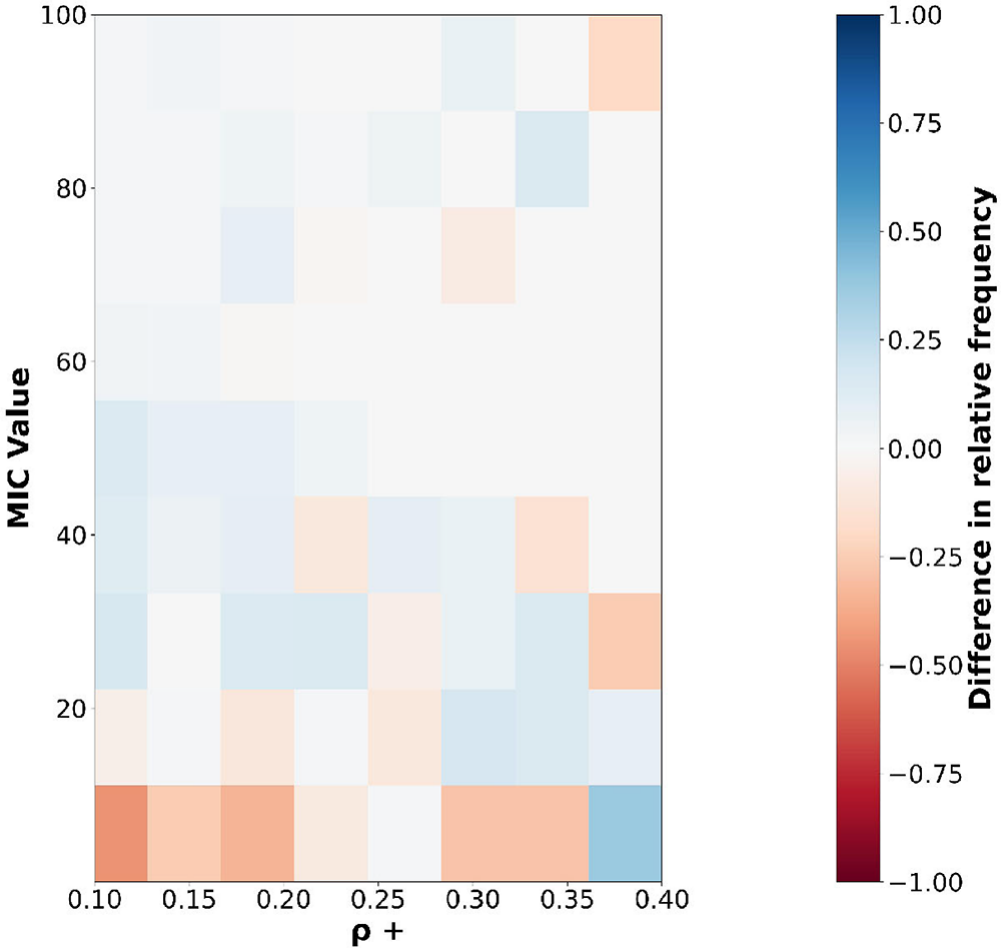
Distribution of poly-R versus poly-K sequences as a function of MIC and charge density (ρ_+_). The heat map shows the difference in relative frequency of occurrence (poly-K minus poly-R) of natural AMPs in a (ρ_+_/MIC) two-dimensional space. Red (blue) boxes imply dominance of sequences containing poly-R (poly-K) motifs, respectively.

For reference, the average values (±SEM) for all natural AMPs used in this analysis, that have an MIC not exceeding 100 μmol/L are peptide length (23 ± 0. 4), peptide charge (3 ± 0.1), charge density (0.148 ± 0.003), and MIC (17 ± 0.9).

### Antimicrobial Activities of the Peptides by Bacterial Susceptibility Tests

We determined the MIC values of four peptides (TP4, TP4-noR5, NP, and NP-R5) against two species of bacteria, the Gram-positive *S. epidermidis* and Gram-negative *E. coli* (Table 4). TP4 was found to be the most efficient against both bacteria. TP4-noR5, which lacks the arginine tail, is less effective than TP4, since four and two times higher concentrations were required for TP4-noR5 to inhibit the growth of *E. coli* and *S. epidermidis*, respectively. Similarly, we find that the terminal poly-R tail enhances the efficacy of NP-R5 compared to NP. While NP shows no antimicrobial properties for the concentrations tested here, NP-R5 shows an efficacy comparable to other AMPs. In separate experiments (Figure S2) using the Gram-negative *Vibrio cholerae*, we found that P1 (FFHHIFRGIVHVGKTIHRLVTG-NH_2_), a natural homologue of TP4 minus the R5 tail, was 16 times less effective than TP4 (MIC of 16 μmol/L for P1 and 1 μmol/L for TP4). This, together with the MIC values in Table 4 and our statistical analysis, indicate that the poly-R motif boosts the effectiveness of these peptides against bacteria.

**Table 4:** Amino Acid Sequence, Molecular Weights, and MIC Values against *E. coli* and *S. epidermidis* of the Studied Peptides^a^

peptides	amino acid sequence	*M*_W_ (g/mol)	*E. coli* (μmol/L)	*S. epidermidis* (μmol/L)
TP4	FIHHIIGGLFSAGKAIHRLIRRRRR-NH_2_	2980.6	4	1
TP4-noR5	FIHHIIGGLFSAGKAIHRLI-NH_2_	2199.7	16	2
NP	QLAQALAAALAALAQGW-NH_2_	1665.9	>64	>64
NP-R5	QLAQALAAALAALAQGWRRRRR-NH_2_	2446.9	7.5	2

a Molecular weights were evaluated using the online software Peptide 2.0 (https://www.peptide2.com/peptide_molecular_weight_calculator.php).

Since bacterial membranes are the main barriers to the entry of foreign molecules, and the major targets for AMPs, the determined MIC values provide the first clues into how the compositions and structures of various bacterial cell membranes may affect the antibacterial potencies of AMPs. For all peptides studied here, the concentrations required to inhibit Gram-negative *E. coli* were higher than those for Gram-positive *S. epidermidis*, hinting to the important structural role of the LPS-rich OM in opposing the permeabilization of Gram-negative bacterial cells by AMPs. To test whether the observed trend is related to differences in permeabilization efficiency of the significantly different bacterial envelopes and lipid compositions of Gram-positive and Gram-negative bacteria, we report on permeabilization assays in the next section.

### Bacterial Membrane Permeabilization Tests

We performed a biological assay based on propidium iodide (PI), a nonpermeabilizing dye that can enter a bacterial cell only when both the outer and inner membranes of the bacteria are compromised through the action of AMPs or cell death. When membranes are permeabilized, the dye can diffuse into the cell, where it can bind to the bacterial DNA, resulting in an enhancement of the fluorescence of the dye.^61^ Likewise, when cells die, their membranes become permeable, and DNA becomes accessible to the dye, regardless of the mechanism of killing.^62^ The fluorescence intensity is therefore used as a reporter on cell death or membrane permeability associated with AMP activity.^63^

Figure 3A,B shows the normalized fluorescence intensities of PI in the presence of *S. epidermidis* and *E. coli*, respectively, treated with the different peptides. The intensities were normalized by the value of fluorescence intensity of PI alone in buffer. TP4 was found to induce the maximum permeabilization in all cases, seen here as the highest relative increase in the fluorescence intensity. At the other extreme, NP was found to cause little or no permeabilization, as the fluorescence intensity in *E. coli* remained close to that of the control. In the order of increasing PI binding, the following trend emerges, NP < NP-R5 < TP4-noR5 < TP4, which is consistent with the MIC values for the bacterial susceptibility assay results in Table 4. We also observe that the PI fluorescence increases on different time scales for the two bacterial species: it saturates quickly for *S. epidermidis* (Figure 3A), but it continues to increase for up to 30 min for *E. coli* (Figure 3B). Taken together, the differences in inhibitory and permeabilization efficacy can be attributed to differences in the membrane structures between the Gram-positive and Gram-negative bacteria^29,64^ and the ability of the peptides to target and bind to those membranes. For the Gram-positive, *S. epidermidis*, the permeabilization rates and efficacies between TP4 and TP4-noR5 are similar, despite a two times lower MIC for TP4 than TP4-noR5. This suggests that, once inside the cell, TP4 may be more effective in binding and disrupting intracellular targets, such as bacterial DNA, than TP4-noR5. For the Gram-negative, *E. coli*, the difference in the permeabilization efficacies is more obvious, and consistent with a four times lower MIC for TP4 than TP4-noR5.

**Figure 3:**
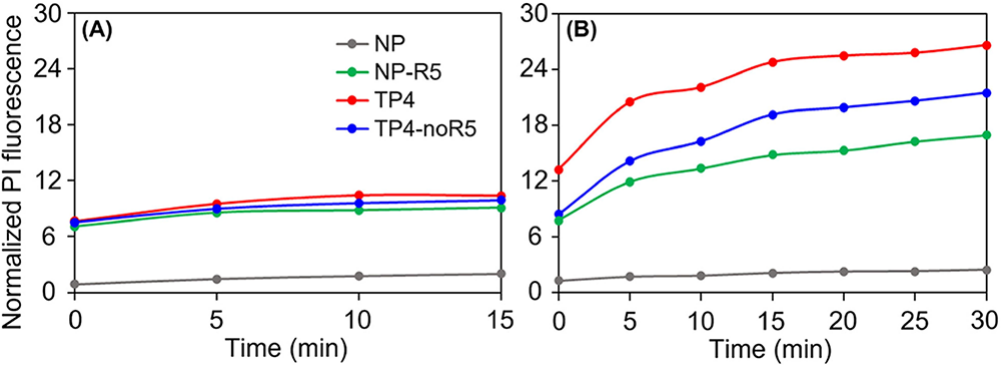
Normalized fluorescence intensities of propidium iodide with time after addition of peptide. (A) *S. epidermidis* and (B) *E. coli*. Error bars represent one standard deviation in the fluorescence intensities from two measurements.

Regarding the different permeabilization rates, one may assume that the thick LPS layer of Gram-negative bacteria hinders OM permeabilization and delay access to the inner membrane. This can, at least partly, explain the slower permeabilization of *E. coli* than *S. epidermidis*. Overall, the R5-containing peptides exhibited stronger permeabilization or bactericidal effects than their counterparts without R5, and are particularly effective against Gram-negative bacteria.

### Calcein Release Assay on Vesicles Exposed to TP4 and Related Sequences

To further investigate permeabilization, as pertains to cytoplasmic bacterial membranes and the role of poly-R motifs, we performed calcein release assays with 3:1 POPC:POPG LUVs used to mimic the charge content of bacteria.^65^ The calcein release assay is a robust method to determine the permeabilization strength of AMPs.^66–68^ The dose–response curves provide the threshold at which a given peptide breaks the bilayer seal, by various mechanisms, leading to leakage. Figure 4 shows the calcein release data obtained when vesicles were exposed to TP4, P1, and P1-R5. We observed sigmoidal curves consistent with a cooperative effect. Table 5 lists the L/P values yielding 50% leakage (EC_50_). TP4 is 2.5 more active (EC_50_ = 71.3 ± 2.6) than P1 (EC_50_ = 28.8 ± 1.3), and adding the R5 motif to P1 to generate P1-R5 (EC_50_ = 97.2 ± 3.4) boosts its activity by a factor of 3.4. Overall, these assays show that the R5 motif plays a major role in the membrane activity of piscidin, as adding it to P1 enables the peptide to perform similarly to TP4.

**Table 5:** Amino Acid Sequence of Peptides Tested via Dye Leakage Assays on POPC/POPG LUVs and Corresponding EC_50_ Values

peptides	amino acid sequence	3:1 POPC/POPG (L/P)^a^
TP4	FIHHIIGGLFSAGKAIHRLIRRRRR-NH_2_	71.3 ± 2.6
P1	FFHHIFRGIVHVGKTIHRLVTG-NH_2_	28.8 ± 1.3
P1-R5	FFHHIFRGIVHVGKTIHRLVTGRRRRR-NH_2_	97.2 ± 3.4

a The standard error is indicated next to the EC_50_.

**Figure 4:**
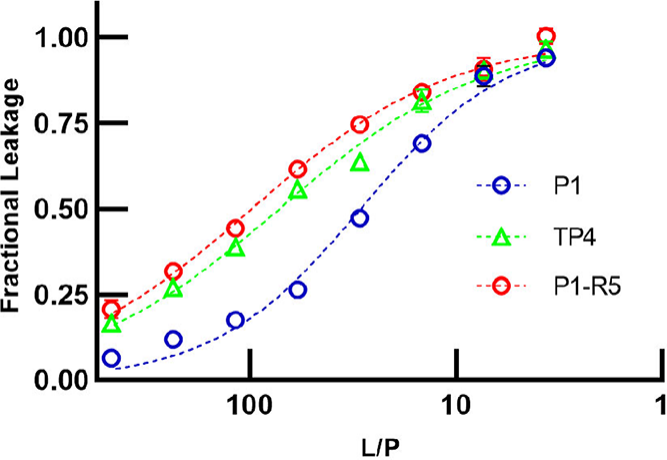
Permeabilization effects of TP4, P1, and P1-R5 on 3:1 POPC/POPG LUVs. LUVs were exposed to increasing amounts of TP4, P1, and P1-R5. The fractional leakage of calcein from the LUVs is plotted as a function of the lipid-to-peptide ratio (L/P). The assays were repeated three times in triplicates. The data shown is the mean ± SD for a representative data set. The error bar, in some cases, is smaller than the symbols used to represent the data points.

### Secondary Structure of the Peptides in Bacterial Membrane Models

CD spectroscopy was used to investigate the secondary structure of TP4 and TP4-noR5 in the presence of LPS-Re representing the first barrier that AMPs are presented with in Gram-negative bacteria. Rough LPS (Re chemotype) contains the minimum number of sugar groups necessary for bacterial survival and plays a critical role in the outer membrane integrity. Given the known heterogeneous chemical nature of LPS,^52^ which increases with the size of the carbohydrate chains, we used LPS-Re (free of lipoprotein) in the hope of a more homogeneous LPS molecular species. Figure 5A shows the CD spectra of pure peptides in buffer, including the control peptides NP and NP-R5. Both TP4 and TP4-noR5 reveal spectral features of random coil structures, while both synthetic peptides NP and NP-R5 display significant α-helical structures, characterized by negative bands at 208 and 222 nm and a positive band at 198 nm.

**Figure 5:**
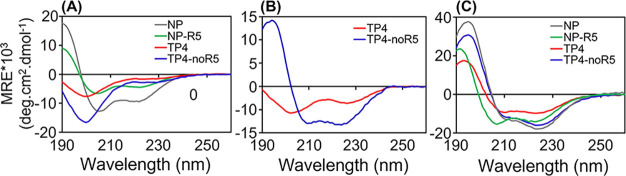
Secondary structure determination in the presence of LPS-Re. (A) CD spectra, showing the mean residue ellipticity (MRE), for four peptides in buffer (10 mmol/L sodium phosphate buffer, pH 7). (B) CD spectra of TP4 and TP4-noR5 in LPS-Re micelles. (C) Same as in (A) for the four peptides in the presence of LPS-Re/POPC (1:5 molar ratio) LUVs. All samples were prepared at 10 μmol/L peptide and 300 μmol/L lipids and measured at 25 °C.

We found that upon binding to LPS-Re micelles, both TP4 and TP4-noR5 experience transitions into structures of low helical content, lower for TP4 than TP4-noR5 (Table 6), as calculated from their mean ellipticities per residue (MRE) (Figure 5B). LPS is known to form micelles in aqueous solution at certain critical concentrations^69^ and can gather into aggregates. We found that the aggregates were difficult to disperse into small, defined objects at reasonable work temperatures. Henceforth, we will refer to these dispersions as LPS-Re micelles.

**Table 6:** Evaluation of the α-Helical Content (% Helicity) of Peptides by CD Spectroscopy^a^

	% helicity		
peptides	buffer	LPS-Re micelles	LPS-Re/POPC LUVs
TP4	0.6	12	25
TP4-noR5	4	21	44
NP	26	n.m.	50
NP-R5	9	n.m.	39

a Peptides were measured in 10 mmol/L sodium phosphate buffer, pH 7 and in the presence of LPS micelles and LPS-Re/POPC LUVs, 1:5 molar ratio. n.m. = not measured.

Both TP4 and TP4-noR5 showed the formation of helical structures, with helical contents of 12 and 21% for TP4 and TP4-noR5, respectively, after interaction with LPS-Re micelles. Because mixtures of LPS-Re and POPC resulted in spectroscopically clearer dispersions, especially after extrusion, they were used here as models for the OM. The fractional helical contents achieved by both peptides increased to 25% for TP4 and 44% for TP4-noR5 when the peptides were exposed to vesicles prepared from a mixture of LPS-Re with POPC, 1:5 molar ratio (Figure 5C). NP and NP-R5 also gain a higher helical content in LPS-Re/POPC relative to buffer (Table 6). The lower helical content in both TP4 and NP-R5, relative to their shorter counterparts (without R5), reflects the contribution from the, presumably unstructured, R5 tail.

### Selective Lipid Binding of the Peptides Using Differential Scanning Calorimetry

Thermodynamic measurements using DSC can offer information on selective binding affinities of AMPs to bacterial membrane lipids from observing specific changes in lipid phase transitions upon AMP addition to a lipid mixture. Preferential interaction with a particular lipid component can cause lipid clustering, a phenomenon believed to be an important mechanism in an AMPs’ action at microbial membranes.^70,71^ We performed DSC measurements to probe the differential interaction of TP4 and TP4-noR5 with LPS-Re/POPE lipid vesicles that mimic the outer membrane of the Gram-negative bacteria and with a mixture of POPE and DPPG that mimics the cytoplasmic membrane.^29,64^

The phase behavior of rough LPS-Re extracted from *Salmonella Minnesota* strain R595 was studied before^72,73^ as a function of temperature, water content, and Mg^2+^ concentration, showing a gel-to-fluid (L_β_ ↔ Lα) acyl chain melting transition temperature (*T*_m_), generally, between 30 and 37 °C. LPS-Re used in this study shows only a very broad transition at ∼55 °C (Figure S3A), which fades with repeated heating–cooling cycles. It is not clear whether this is a chain melting transition or a type of morphological change, but previous X-ray diffraction studies showed that LPS-Re can display a rich morphological behavior that includes hexagonal II or cubic phases at >50 °C.^73,74^ Further, LPS is known to harbor structural heterogeneity that results from biosynthesis and extraction conditions, starting with the basic unit of LPS (Lipid A).^52^ Therefore, we added POPE for our DSC measurements, a well-studied zwitterionic lipid, which shows a gel-to-fluid transition at ∼25 °C. LPS-Re/POPE gives a single transition peak, which indicates that a single, homogeneous phase was obtained when mixing.

Figure 6A shows the transition peaks for LPS-Re/POPE 1:5 in the absence and presence of TP4 and TP4-noR5. The shift toward lower temperature coupled with a broadening of the transition (both more pronounced for TP4-noR5 than TP4) indicates that TP4-noR5 is more disruptive to lipid packing, consistent with a deep partitioning into the acyl chains. The lesser shift observed with TP4 indicates a weaker interaction, consistent with the previously postulated surface binding of TP4 to the headgroups via the poly R5 tail. The evolution of those changes is clearer in the raw data (Figure 6B,C). A split peak pattern is identifiable in the repeated cooling scans, evolving in opposite directions for TP4 and TP4-noR5, indicating the creation of distinct microenvironments upon peptide binding, consistent with previously observed preferences of AMPs for a subset of lipid components or induced phase separation.^71,75,76^ Similar effects were observed in POPE/DPPG mixtures, which show split transition peaks consistent with phase separation when peptide is added (Figure 6D–F). Overall, albeit qualitative in nature, the DSC data show that TP4-noR5 has a higher capacity for chain melting and mixing, while TP4 may interact more favorably with LPS-Re and DPPG than POPE, through the action of the charged R5 tail on the lipid headgroups. To further explore this hypothesis, we performed solid-state NMR studies of oriented TP4-bilayer samples.

**Figure 6:**
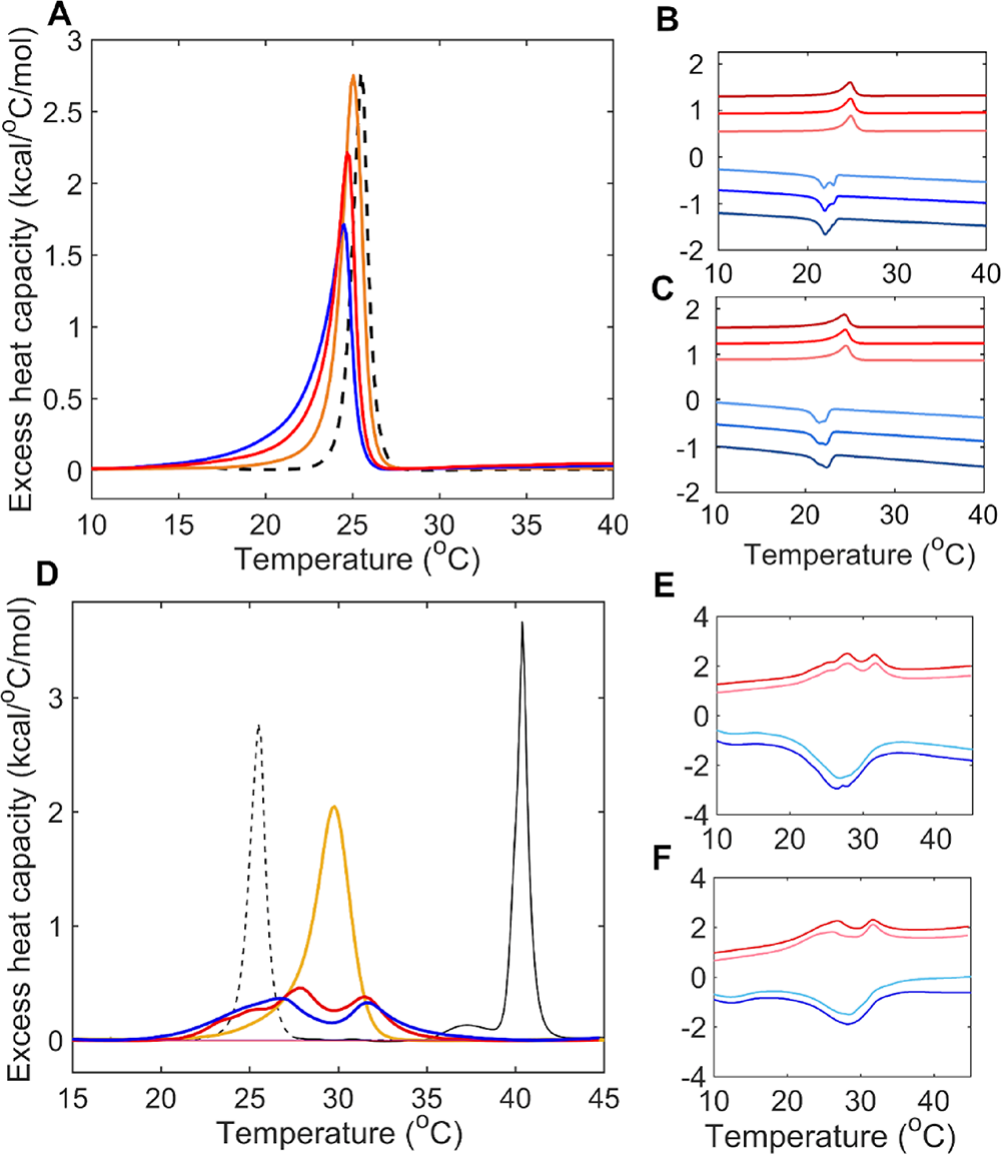
Differential scanning calorimetry of LPS-Re/POPE and POPE/DPPG with TP4 and TP4-noR5. (A) Heating scans for pure POPE (black-dashed, *T*_m_ = 25.5 °C), LPS-Re/POPE 1:5 molar ratio (orange, *T*_m_ = 25.0 °C), TP4-noR5 in LPS-Re/POPE (blue, *T*_m_ = 24.5 °C), and TP4 in LPS-Re/POPE (red, *T*_m_ = 24.7 °C). The third heating scans are shown here, from a series of consecutive heating/cooling scans. (B) Raw DSC scans for LPS-Re/POPE (molar ratio 1:5) with TP4. Three consecutive heating (red) and cooling (blue) scans were recorded (in the order from light to dark shades) and were shifted from each other along the vertical axis for better visibility. (C) Same as in (B) for TP5-noR5. All samples were prepared in water at P/L 1:50 molar ratio. Lipid concentration was 2.08 mmol/L in the case of pure lipid solution and 1.04 mmol/L in the presence of peptide. The scan rate was 0.5 °C/min. (D) Second heating scan for POPE/DPPG at 1:1 molar ratio (yellow) and in the presence of TP4 (red) and TP4-noR5 (blue), at P/L of 1:50. The spectra for POPE (dashed, black) and DPPG (solid black, *T*_m_ = 41 °C) are overlaid. (E) Raw DSC heating and cooling scans for POPE/DPPG with TP4. (F) Same as in (E) for TP5-noR5. All samples were prepared in water at a lipid concentration of 3.4 mmol/L.

### Solid-State NMR Studies of Oriented TP4-Bilayer Samples

Oriented sample solid-state NMR (OS SS-NMR) was employed to investigate the effect of TP4 on the membrane organization as well as the structural features of the peptide. Samples featuring aligned 3:1 POPC/POPG bilayers were prepared, and the ^15^N-Gly13 labeled TP4 peptide incorporated at P/L = 1:60 and 1:30. Due to its high abundance in phospholipid headgroups, ^31^P is readily detected. ^31^P OS SS-NMR provides useful insight into peptide-lipid interactions.^77–83^ As shown in Figure 7, neat POPC/POPG bilayers display two nonfully resolved ^31^P resonances following the expected 3:1 ratio and resonance values of 29 and 26.5 ppm for POPC and POPG, respectively.^84^ A minor signal appears near −15 ppm, corresponding to unaligned lipids on the edge of the glass plates. When the TP4 concentration is increased, the POPG resonance moves upfield while that of POPC is unaffected. Since the PC headgroup is more sensitive than PG to the membrane surface charge that occurs when charged molecules are added,^85^ the lack of response from PC strongly supports the conclusion that TP4 mostly interacts with the PG headgroups.

**Figure 7:**
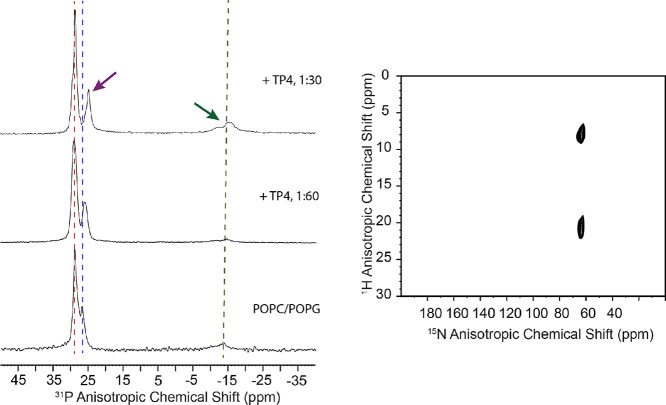
Solid-state NMR studies of oriented TP4-bilayer samples. Left: TP4 was incorporated in aligned POPC/POPG bilayers at P/L = 1:60 and 1:30, and the ^31^P NMR data were collected at a frequency of 242.9 MHz, with the bilayer normal parallel to the static magnetic field. The red and blue lines correspond to the POPC and POPG signals in the lipid-only sample, respectively, while the green line highlights the area of the spectrum consistent with lipid headgroups adopting a disrupted orientation. The purple arrow points at the POPG signal, which is shifted upfield by TP4, while the green arrow features the increased signal in the region associated with disrupted headgroups. Right: A 2-D HETCOR solid-state spectrum is shown for TP4 in the sample prepared at P/L = 1:60. The peptide was ^15^N-labeled at Gly-13.

Additionally, the signal at −15 ppm increases when the P/L changes from 1:60 to 1:30, consistent with increased disorder in the headgroup region when the peptide concentration increases. We note that the samples of POPC/POPG and POPC/POPG + TP4 at P/L = 1:60 appear to have a similarly small signal at −15 ppm, albeit the lower signal-to-noise ratio for the lipid only sample prevents a more definitive conclusion. A small amount of unoriented sample could be present due to edge effects on the glass. We also used ^15^N OS SS-NMR to characterize the peptide structure and orientation in the same samples used for the ^31^P NMR experiments. We collected a 2D HETCOR spectrum on ^15^N-Gly13 TP4 at P/L = 1:60 to access two important structural restraints, the ^15^N backbone chemical shift and the ^15^N–^1^H dipolar coupling.^86,87^ As shown in Figure 7, an excellent spectrum was obtained, Gly13 resonates near 65 ppm, with a dipolar coupling of 10.5 kHz. These results are consistent with an α-helical structure that sits parallel to the membrane surface, as previously detected for P1.^50–52^

### Structures of Peptide/Bilayer Complexes Studied with X-ray Diffraction

The LPS-Re used in these studies did not show any lamellar or other forms of long-range, organized structure by itself, which suggests that it is heterogeneous and forms amorphous aggregates or micellar structures.^69^ On the other hand, LPS-Re could be homogeneously incorporated into phospholipid bilayers such as POPC to obtain strongly diffracting lamellar stacks from which electron density profiles could be calculated (Figure 8A), based on structure factors with phases determined by the swelling method (Figure S4A–C, Table S6).

**Figure 8:**
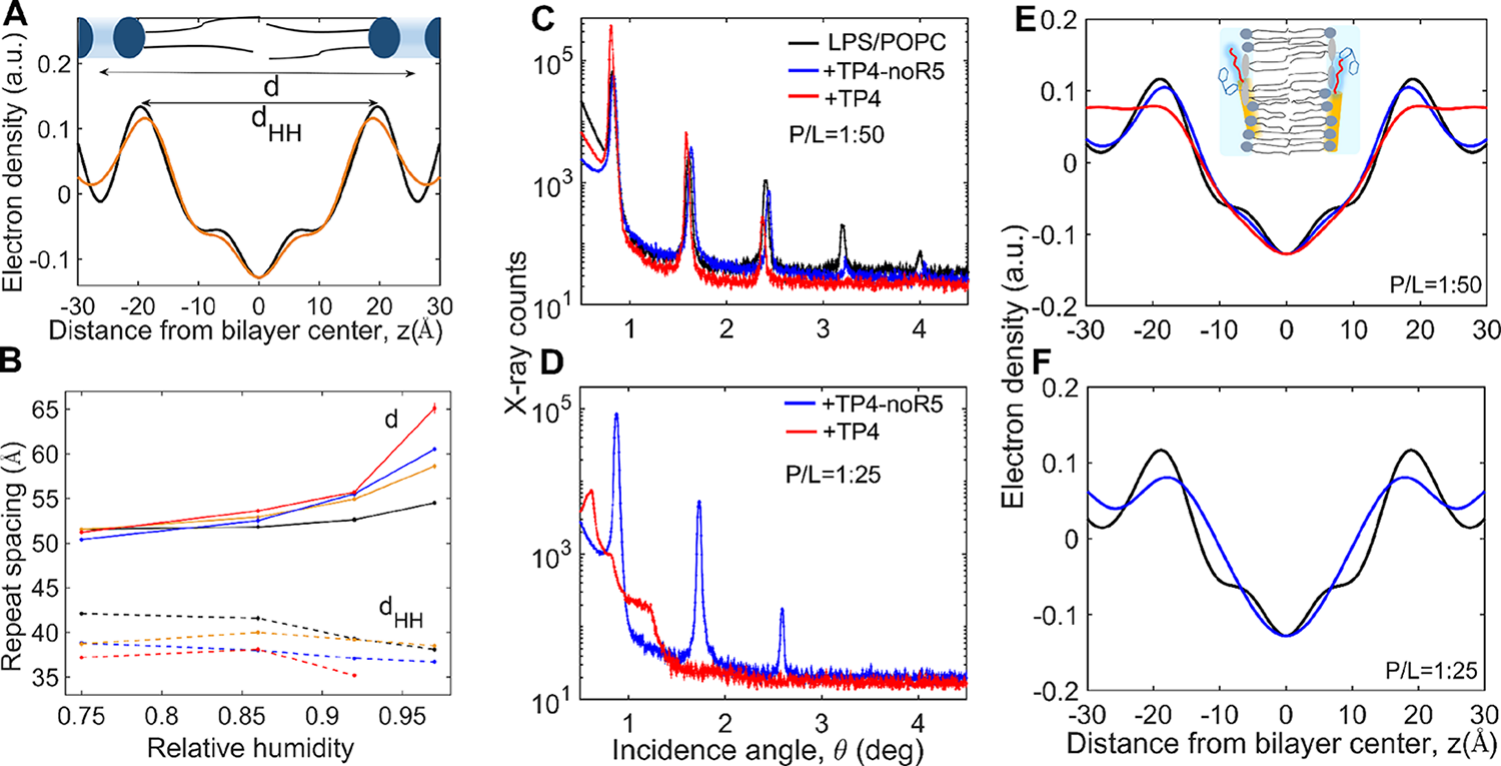
X-ray diffraction data from oriented multilayer (lamellar) samples of LPS-Re/POPC with TP4 and TP4-noR5. (A) Electron density profiles of a bilayer from a lamellar sample of POPC (black) and LPS-Re/POPC, 1:5 molar ratio (orange). The repeat distance (*d*) includes the bilayer thickness and the water of hydration (Figure 1). The electron density profiles are shown on an arbitrary scale, with amplitudes adjusted to match in the middle of the bilayer (*z* = 0) for an easier comparison. (B) Variations in the repeat distances (*d*) and headgroup-to-headgroup distances (*d*_HH_) as a function of relative humidity (RH) for lamellar samples made of POPC (black), LPS-Re/POPC (orange), LPS-Re/POPC + TP4-noR5 (blue), and LPS-Re/POPC + TP4 (red). All samples were produced from LPS-Re/POPC (1:5), at P/L = 1:50 and measured at 25 °C and various hydrations. *d*_HH_ for LPS-Re/POPC + TP4 at 97% RH could not be calculated unambiguously due to broadened Bragg peaks (Figure S4D). (C) Diffraction data from lamellar samples of LPS-Re/POPC (black), LPS-Re/POPC + TP4-noR5 (blue), and LPS-Re/POPC + TP4 (red), for P/L = 1:50. (D) Same as in (C) for P/L = 1:25. (E) Bilayer electron density profiles (± peptides) were calculated from the data in (C) and Table S7 (the inset is a cartoon interpretation of the bilayer/peptide assembly, showing a bilayer with peptide bound to surfaces shown as yellow cylinders and hydrated R5 segments in red). (F) Same as in (C) for P/L = 1:25. A bilayer density profile could not be calculated for LPS-Re/POPC + TP4 from the distorted diffraction signal. Profiles shown here are for 93% RH and 25 °C.

Of note is the slight relative increase in the density in the flanks of the POPC bilayer in the presence of LPS-Re (Figure 8A). This is due to the sugar groups and associated water protruding outward, past the lipid phosphate line. This increase in density at the bilayer surfaces is accompanied by an increase in the repeat spacing (*d*) of the lamellar stack. The increase is most noticeable at higher hydrations and in the presence of peptides, especially TP4 (Figure 8B, top) and is due to the additional water accrued by the sugar moieties of LPS-Re and by the peptides bound to bilayer surfaces. In contrast to *d*, the headgroup-to-headgroup distance (*d*_HH_), which broadly defines the hydrocarbon region thickness, shows a slight decrease with hydration and peptide addition (Figure 8B, bottom). These changes are the first indicators of the effects of membrane-active AMPs partitioning in bilayers and exhibiting, in some cases, opposite and concomitant effects, such as hydrocarbon thinning and swelling due to water retention. Both peptides produce an increase in *d*, concomitant with a decrease in *d*_HH_, compared to both POPC and POPC/LPS-Re. However, all changes are much more pronounced for TP4 than TP4-noR5 and more noticeable for higher hydrations (Figure 8B). For instance, at 97% RH and a P/L = 1:50 molar ratio, *d* reaches 65.1 Å for LPS-Re/POPC in the presence of TP4, compared to 60.5 Å for TP4-noR5 and 58.6 Å for LPS-Re/POPC without peptides.

For a better comparison of the bilayer structural effects caused by TP4 and TP4-noR5, the two peptides were incorporated into LPS-Re/POPC bilayers at various P/L ratios and the electron densities of the bilayer/peptide complexes were constructed, where possible. At a P/L of 1:50, the density profiles (Figure 8E) reveal only slight perturbations to the LPS-Re/POPC bilayer structure in the presence of TP4-noR5. Interestingly, the profile with TP4 shows similarly small perturbations in the hydrocarbon region, at this P/L, but a significant density increase at the bilayer boundaries and in between adjacent bilayers. The pronounced increase in *d* and rise in electron density at the bilayer surfaces for TP4, but not for TP4-noR5, indicates that the R5 tails of TP4 populate the space at the surfaces of LPS-Re/POPC bilayers, where it interacts with the LPS carbohydrate and the lipid phosphate groups and also recruits significant amounts of water.

Increasing P/L from 1:50 to 1:25 causes a dramatic bilayer disruption (Figure 8D), seen here as a loss of lamellar diffraction signal for TP4, but not for TP4-noR5. This is consistent with the fact that strong membrane perturbation effects, often associated with permeabilization, are detected only above a certain P/L threshold, here ∼1:25. The preferential interactions of TP4 with a particular lipid component, here LPS-Re, can cause a slew of effects, such as separation of lipids in domains of different thicknesses and hydrations and morphological bilayer transformations, seen here as a loss of lamellar diffraction signal (Figure 8D). At this higher P/L, TP4-noR5 itself shows a significant broadening and shift of the profiles maxima inward as the bilayer thins, relative to the unperturbed bilayer (Figure 8F). Taken together, XRD data reveals that the N-terminal hydrophobic segment and the C-terminal R5 tail act in concert to disrupt the bilayer barrier, through different interactions with the hydrocarbon and the surface-exposed molecular groups of the multicomponent bilayer.

We conducted further investigations into the interactions of these peptides with POPE/POPG (3:1), a mixture that mimics the lipid composition of the inner bacterial membranes.^88^ When measured at 30 °C, above the gel–fluid phase transition temperature of POPE, the POPE/POPG (3:1) mixture displays one set of equidistant Bragg peaks (signature of a homogeneous blend, in a single lipid phase) and up to eight orders of diffraction (Figure 9A). Both TP4 and TP4-noR5 alter the diffraction pattern, but their effects are significantly different. TP4 shows pronounced broadening of the diffraction peaks and an increase in the repeat distance by approximately 10 Å relative to the neat POPE/POPG bilayer. By contrast, TP4-noR5 causes a very large decrease in the repeat spacing, by ∼5 Å in both *d* and *d*_HH_ (Figure 9A), also seen as an inward shift of the headgroup regions in the electron density profile (Figure 9B). In an attempt to isolate the effect caused by the R5 tail, we investigated the bilayer (POPE/POPG) interactions with the inert, helical peptide NP, and its poly-R variant, NP-R5. NP shows minimal perturbations to the diffraction signal (Figure 9A inset, blue), while the resulting bilayer profile (Figure 9B) indicates superficial binding at best, as previously reported for this peptide^27^ and observed here as a slight broadening and shift of the profiles maxima outward, with no thinning of the hydrocarbon region. This is expected if small amounts of NP peptide attach to the bilayer surface without significant insertion. In contrast, NP-R5 displays a pronounced broadening of the diffraction signal (Figure 9A inset, red)**,** indicative of lipid segregation effects as seen for TP4.

**Figure 9:**
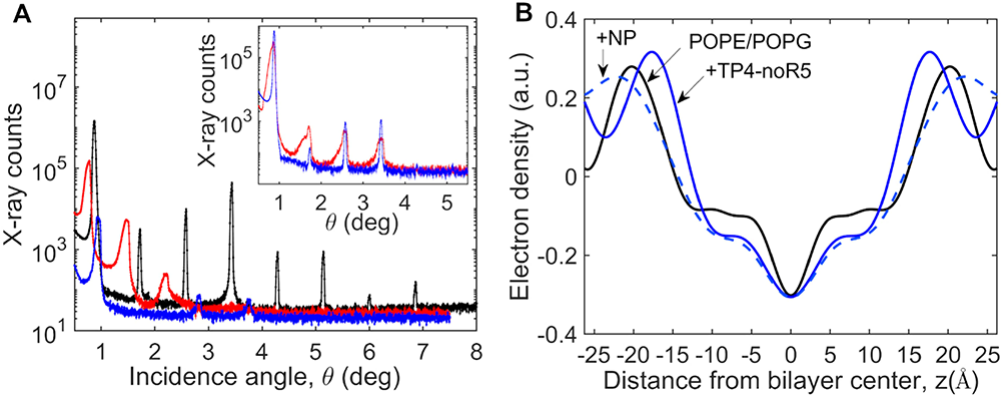
X-ray diffraction data from oriented multilayer samples of POPE/POPG with peptides. (A) Diffraction data for POPE/POPG 3:1 without peptide (black), with TP4-noR5 (blue) and with TP4 (red), at P/L was 1:2. Samples were measured at 97% RH and 30 °C. The repeat spacing, *d*, the headgroup-to-headgroup distances, *d*_HH_, and their uncertainties (one standard deviation) are given in Angstrom: neat POPE/POPG (black, *d* = 51.7 ± 0.1; *d*_HH_ = 40.5 ± 0.1), with TP4-noR5 (blue, *d* = 47.2 ± 0.4; *d*_HH_ = 35.4 ± 0.5), and with TP4 (*d* = 61.4 ± 1.3). Inset: the same POPE/POPG system, with NP (blue, *d* = 55.1 ± 0.1; *d*_HH_ = 44.3 ± 0.2) and with NP-R5 (red). (B) Electron density profiles calculated from diffraction data shown in (A), respectively. The profiles for the TP4 and NP-R5, and hence, values for *d*_HH_ could not be unambiguously determined due to the pronounced deformations of the peaks. See also Figure S5A for other measurement conditions. The amplitudes of the profiles were adjusted by arbitrary scale factors until they matched at *z* = 0 for an easier comparison. Structure factors are given in Table S8.

Overall, the XRD results indicate that the disordered poly-R tail has a separate role from the helical, hydrophobic body of the AMP in their action at membranes. In particular, the R5 tail in TP4 exacerbates preferential interactions with LPS-Re and PG groups at bilayer surfaces, causing vulnerable boundary regions and membrane ruptures due to lipid segregation in domains of different thicknesses and hydrations. This can predispose the bacterial membranes to the attachment and insertion of hydrophobic, helical segments into the exposed acyl chains, as a prerequisite for pore formation.

### Neutron Reflectometry of TP4-Bilayer Complexes

Neutron reflectometry is an interfacial scattering technique that reveals the structure of bilayers adsorbed at solid interfaces. Neutrons have good contrast between lipid acyl chains and proteins, such that the individual components—protein, lipid acyl chains, and lipid headgroups—of a protein/bilayer complex can be discriminated. Neutron reflectometry data are shown in Figure S6, while Figure 10 shows the fractional volume occupancy of each of the components in a DOPE/POPG (3:1 molar ratio) bilayer adsorbed on natural oxide-terminated silicon (Figure 10A,B) and subsequently exposed to TP4 (Figure 10C) and TP4-noR5 (Figure 10D).

**Figure 10:**
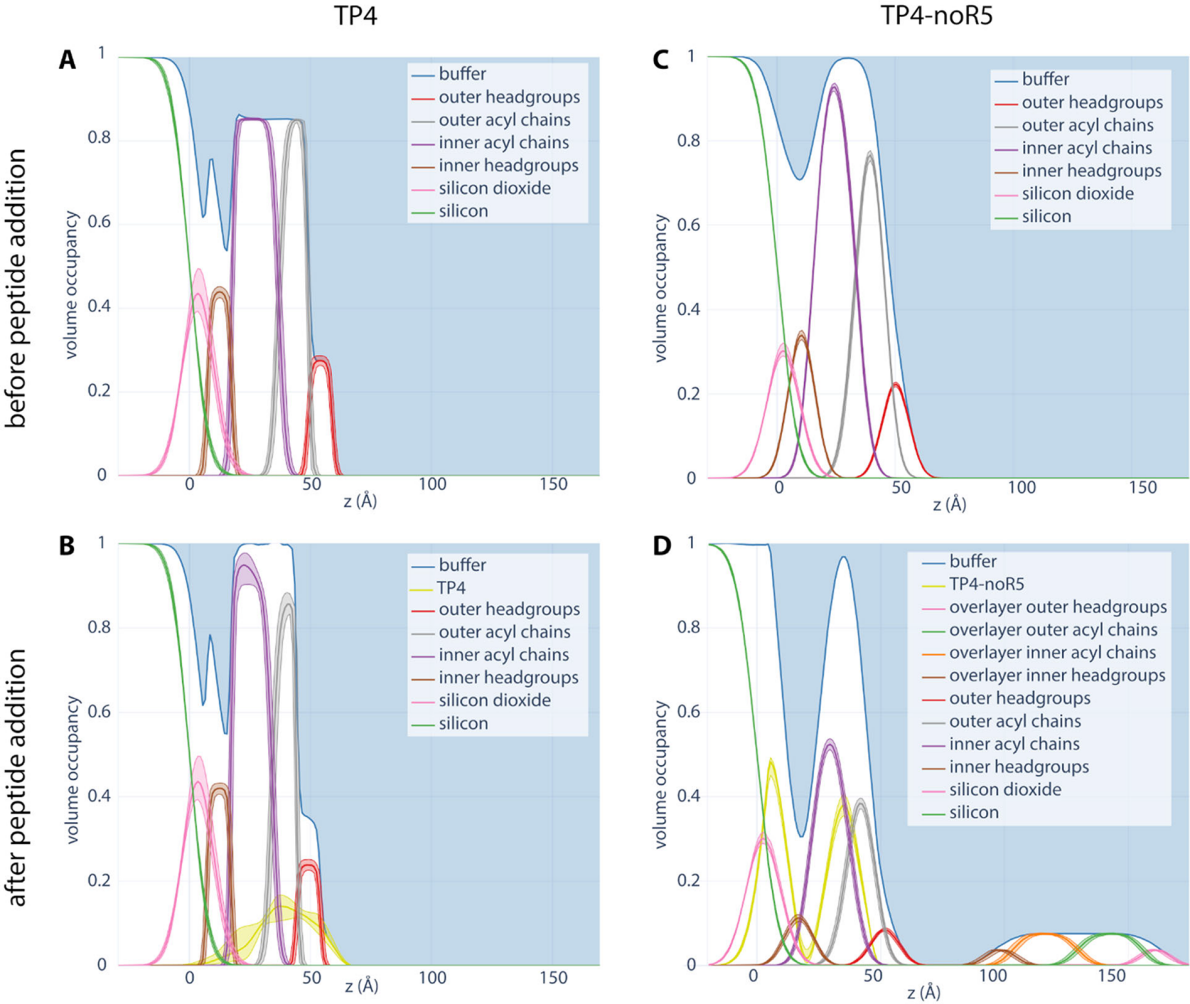
Volume occupancy distributions representing the structure of the bilayer/peptide complex following the exposure of DOPE/POPG (3:1) lipid bilayers adsorbed on silicon wafers (A, B) to TP4 (C) and TP4-noR5 (D) dissolved at 3 μmol/L in 10 mmol/L tris buffered at pH 7.4.

Full-length TP4 is found throughout the bilayer structure, with (58 ± 5)% (68% confidence) found in the acyl chains and (29 ± 2)% (68% confidence) in the headgroups and the balance in the buffer. TP4 causes significant thinning of (4.1 ± 0.2) Å (68% confidence) of the hydrophobic region. This is sufficient to increase the bilayer completeness from about 85% without peptide to almost 98% with peptide. The excess lipid appears to form multilamellar structures that appear as a peak near *Q*_z_ ≈ 0.13 Å^–1^, which is excluded from the analysis (Figure S6). Using the periodictable software (https://github.com/python-periodictable/periodictable), the peptide volume is estimated to be 3955 Å^3^, based on the sum of the volumes of the individual amino acids, and the molecular weight is as in Table 4. The integrated protein density profile is 318_–35_^+42^ Å^3^ per lipid area (about 72 Å^2^ averaged over the two leaflets), for a total lipid/protein ratio of about 12 across the two leaflets.

TP4-noR5 causes a similar thinning effect but has its density concentrated in the hydrophobic region of the bilayer. Following exposure to TP4-noR5, the volume occupied by lipids at the surface is reduced by about half; the excess lipids appear as a thick incomplete overlayer above the original disrupted bilayer, as well as in a more pronounced multilayer peak around *Q*_z_ ≈ 0.13 Å^–1^ that was also removed from the NR pattern before analysis. We observe some TP4-noR5 at the silicon dioxide surface underneath the lipid bilayer. The dimensions of this layer match those of a control experiment with TP4 on a bare silicon dioxide surface (Figure S7). This finding suggests that TP4-noR5 may penetrate the hydrocarbon core of the lipid bilayer to a greater extent than full length TP4.

## Discussion

The increasing availability of sequences of AMPs (both natural and synthetic), in vitro antimicrobial activity parameters, and biophysical data, allows for examining the correlations between AMP sequences and their bioactivities and identifying important sequence motifs that endow natural AMPs with selectivity and potency in the environment displayed by bacterial membranes. Fish AMPs, for example, are rich in motifs that make them extremely effective bacterial killers. These include the presence of the ATCUN motif that binds and transports the ROS-promoting Cu^2+^/Ni^2+^ ions into the bacterial cells; the broken helix at G13, which allows the two halves to maximize their hydrophobic moment for maximum insertion into the bilayer hydrocarbon^89–91^ and, for several of them, a cluster of R residues at the C-terminus.^10,11,19^ The poly-R motif is rather elusive among the natural AMPs identified or cataloged in databases. However, it is prevalent in CPPs. The TAT protein transduction domain (PTD) of the human immunodeficiency virus (HIV-1), an arginine-rich, naturally occurring CPP^92^ has served as a model for both CPP and AMP design. For instance, the addition of a cluster of arginines (R2, R8 and dendrimeric R) to Vancomycin was shown to improve its antimicrobial profile and help with combating bacterial resistance.^17,18^,^93^ Thus, it is interesting and important to explore the functionality of the poly-R segments in CPPs and AMPs, from a structural perspective.

In the following sections, we discuss how: (1) AMP database analysis and experiments on specific peptides (NP, TP4, P1) confirm bactericidal efficacy conferred by poly-R segments; (2) poly-R affects the structures of the lipids and peptides; and (3) possible models of interaction of poly-R with lipid membranes that can reconcile structural and functional observations.

### Polycationic Motifs Increase Bactericidal Efficacy

The importance of clustered cationic residues in natural AMPs and in particular, poly-R motifs is established in several studies of AMPs from all kingdoms of life.^10–16,19,21^ For instance, hLF(1–11), a highly efficient antibacterial^15^ and antifungal^94^ peptide derived from human lactoferrin, carrying four R residues in the N-terminal side, was found to be less effective in the killing of bacteria^95^ and showed decreased binding to bacterial lipopolysaccharide^96^ when those N-terminal residues were removed.

Similarly, in this work, removing the poly arginine (R5) C-terminal segment from the natural TP4 peptide causes a reduction in antimicrobial efficiency, seen here as a 4-fold increase in MIC values against the Gram-negative *E. coli* and a 2-fold increase against the Gram-positive *S. epidermidis* (Table 4). P1, lacking the R5 tail, also shows a 16-fold lower antimicrobial efficacy against *V. Cholerae* than TP4. As an additional control, simply adding an R5 segment to a broadly inert peptide (NP) imbues the construct with strong antimicrobial properties (Table 4). Interestingly, despite the fact that TP4 contains a more strongly amphipathic, hydrophobic and helical N-terminal segment than NP-R5, the MIC is only 2 times lower for TP4 than NP-R5. Propidium iodine uptake reveals that bactericidal efficiency follows the same pattern as with MIC values: NP < NP-R5 < TP4-noR5 < TP4, with the neutral peptide showing almost zero permeabilization. Clearly, both segments of TP4 (the amphipathic N-terminal body and the disordered poly-R, C-terminal segment), through their very different chemical nature, have separate roles in bacterial membrane interactions and permeabilization. We assessed those functions through structural measurements.

The database analysis of AMPs containing polycationic motifs confirms that this result can be generalized across a wide range of peptide sequences (Tables 1–3). The widespread in MIC values observed from naturally occurring AMPs, even with similar polycationic motifs, suggests that the mechanism of action of the polycationic motif is synergistic with the uncharged regions of the peptide.

### Structural Consequences of the Poly-R Motif at Model Membranes

CD measurements of the secondary structures of four investigated peptides reveal that, when exposed to membranes, the -R5 variants are less helical, overall, than the -noR5 counterparts (Figure 5), but have stronger antibacterial activities (Table 4). Thus, the helical content of these AMPs does not necessarily correlate with antimicrobial efficacy. By extrapolation from the high-resolution NMR structure of piscidin P1,^89^ which is almost completely helical in lipid bilayers, the lower helical content (per residue) of an -R5 variant is most likely due to the contribution from the disordered poly-R segment. Interestingly, studies of lysine-rich peptides showed that peptide constructs that are not prone to aggregation when bound to LPS, and concomitantly display larger unstructured regions, highly favor LPS permeabilization.^97^ By analogy, the highly charged and disordered R5 can reduce the chance of aggregation at surfaces, thus facilitating peptide navigation through the LPS and access to the hydrocarbon core. Indeed, our CD data show that TP4 maintains a flexible, mostly disordered, conformation outside and inside LPS-Re. The helical content expands in a mixture of LPS-Re/POPC. This increase can be explained considering that, once past the LPS-Re polysaccharide groups, the peptide has immediate access to a (POPC) bilayer-water interface, which is a trigger to the “partitioning-folding” phenomenon^98^ for amphipathic helices. In the case of TP4, once recruited to the anionic surfaces through the Coulombic attraction of the R5 segment and hydrogen bonds, the hydrophobic, helical segment of the peptide can then be expected to insert into the hydrocarbon region of the membrane promoting further destabilization.

Evidence for the importance of recruitment of peptide to the bilayer-water interface, as well as for insertion of the peptide into the hydrocarbon region, is present in the DSC, NMR, and NR data. The DSC results suggest that the presence of peptide can lead to chain melting and mixing in an LPS-Re/POPE mixture (Figure 6A–C); in fact, TP4-noR5 appears to be more effective than full length TP4, assuming that the quantity of TP4 on the surface is equal to or exceeds that of TP4-noR5. In POPE/DPPG, DSC provides evidence of phase separation (Figure 6D–F). This can be attributed to the preferential interaction of TP4 with the PG headgroups, which is confirmed by NMR in POPC/POPG (3:1) mixtures (Figure 7). These results are consistent with the NR results on DOPE/POPG mixtures, which show that both peptides reside in the bilayer and, to a large extent, in the hydrocarbon region (Figure 10). TP4 displays a broad, continuous distribution across hydrocarbon and outside the outer leaflet surface. TP4-noR5 shows narrower distributions, one in the acyl chains and one at the substrate surface, indicating that TP4-noR5 can translocate more easily across the hydrocarbons.The strong association of the R5 segment with the PG headgroups on the surface, coupled with the opposite tendency of the hydrophobic segment to immerse and translocate though the hydrocarbon, may impose greater stress and a richer morphological landscape of the TP4-bilayer complex, then any of the two segments taken separately.

The XRD results on multilayer stacks provide further structural detail (Figures 8 and 9). Overall, the signals from bilayer stacks were strongly distorted by NP-R5 and TP4, the constructs containing poly arginine motifs. Some of these distortions, which manifests as a strong increase in lamellar spacing and water layer thickness and broadening of the peaks, may arise from salt effects. Assuming the area density of TP4 observed in the NR experiments (one molecule per 12 lipids at 72 Å^2^ per lipid), the effective concentration of TP4 bound to two leaflets enclosing an interlamellar spacing of 10 Å is about 0.4 mol/L. Because TP4 has a +7 charge, the effective ionic strength is above 1 mol/L. It has previously been observed that even at mmol/L salt concentrations, the salt environment of charged bilayers has a strong effect on the multilayer adhesion^99–102^ and, under some circumstances, can lead to unbinding of lamellae.

Peak broadening can also arise from peptide-induced lipid segregation. At temperature close to lipid phase transitions, a lipid phase separation can occur concurrent with lipid segregation for which there is evidence in the DSC and XRD results (Figures 6, 8D and 9A). This can occur if the peptide preferentially attaches to one of the lipid headgroups, causing the formation of a domain enriched in that headgroup across multiple layers. In this case two sets of peaks, each with its own repeat spacing, contributes to the distorted diffraction peak. For POPE/POPG (3:1) bilayers, for example (Figure 9), peak broadening may indicate the formation of TP4/PG-rich domains, separate from POPE-rich domains, across multiple bilayers. At temperatures ∼25 °C, the segregated POPE can exhibit a gel phase characterized by a thicker bilayer. TP4-noR5 does not produce the broadening effect in POPE/POPG bilayers, suggesting that any domain formation is charge-driven. Interestingly, the broadening effect is not observed for POPC/POPG (3:1) bilayers (Figure S5B) with either peptide; this may be due either to a higher selectivity of TP4 for POPG relative to POPE than to POPC, or a higher propensity for phase separation between POPE and POPG than between POPC and POPG.

In both POPE/POPG (3:1) and POPC/POPG (3:1) bilayers, TP4-noR5 decreases the repeat spacing (by ∼5 Å in both *d* and *d*_HH_). A bilayer thinning effect, due to the immersion or intercalation of the peptide body between lipid headgroups, causing a bilayer area expansion at constant hydrocarbon density, has often been observed with AMPs,^41,103^ but not to this extent. Notably, NP does not thin the bilayers. Hydrophobicity and the degree of separation into hydrophobic and hydrophilic sides around the folded peptides (hydrophobic moment) influence the depth of insertion of a hydrophobic segment. Notably, TP4-noR5, just like P1, contains multiple membrane-anchoring phenylalanines that promote a strong hydrophobic interaction with the bilayer hydrocarbon core and a high hydrophobic moment. These features are less pronounced in the alanine-rich NP variants, which could be the reason for the reduced bilayer thinning and, ultimately, their significantly lower antibacterial efficacy than TP4 variants.

Our DSC and X-ray diffraction data, taken together, indicate that the poly-R segments dominate the initial interactions with membrane surfaces as they target PGs, LPS-Re (and less so, PEs). Once established at the membrane surface, they can induce morphological transformations and lipid segregations in domains or phase separations. At the boundaries between such domains, the hydrocarbon region may become exposed through, e.g., curvature and hydrocarbon thickness mismatches. This can facilitate anchoring of the amphipathic, helical peptide segment in the membranes, as a prerequisite for pore formation.

Clustering of anionic lipids with AMP, through preferential interactions, has been argued to be an important mechanism of membrane disruption and bacterial killing,^71,104^ and we see here that it is mainly charge driven. The stronger effects of TP4 on mixed bilayers containing LPS-Re or POPG, in comparison to TP4-noR5, is most clearly seen in the X-ray diffraction data, which reveals drastic morphological changes occurring in LPS-Re/POPC or POPE/POPG bilayers in the presence of TP4, culminating with an almost complete loss of lamellar signal at a P/L ratio of 1:25 (Figures 8D and 9A). While it is difficult to assess the identity of the newly formed structures, both micellization of the LPS-Re under the effect of TP4,^20^ and nonlamellar structures are likely to occur.^97^ Electron microscopy images captured such a micellization process of the OM of Gram-negative bacteria *H. pylori*, under the action of TP4 at less than 1 μmol/L concentrations.^20^

The concentration ranges needed to inhibit bacterial growth (MIC values) in culture media is expected to be correlated with the P/L ratios at which permeabilization is detected in model membranes. In previous studies, addition of P1 at 3 μmol/L resulted in a P/L in the range of 1:10 to 1:8 of tightly bound peptide helices and complete surface coverage in model bilayers.^90,91^ Also, a P/L of 1:10 corresponds to 100% calcein leakage from POPC/POPG liposomes treated with TP4, P1-R5 and ∼75% with P1 (Figure 4). In a culture broth, we expect that much of the AMP molecules are sequestered by binding to various broth components, making it difficult to determine the actual fraction of peptide that reaches the bacterial membrane. It would appear as if larger concentrations are needed to efficiently inhibit bacteria. We can think of a few reasons why this is not the case for TP4 or P1, which show MIC values of <1 μmol/L. First, the concentration of the peptide accrued at membranes will be orders of magnitude greater than that in bulk solution,^105^ probably reaching the surface coverage sufficient for membrane destabilization. The anionic bacterial membranes act as effective concentrators. Second, the most potent piscidins can penetrate and inflict significant damage to bacteria, by interfering with normal cellular processes, including membrane protein function,^91^ at well below the concentrations needed for complete coverage.^106^ This could happen through various mechanisms including endocytosis, direct translocation, and transient, local pore formation.^107,108^ In any case, membrane destabilization, which is usually seen at high P/Ls in model membranes,^8^ is aided by AMPs creating and exploiting membrane heterogeneity (e.g., regions of high curvature stress and domain boundaries) for entry.^91^

As we discover here, the R5 segment of TP4 is the main promoter of segregation in mixed anionic-zwitterionic lipid systems specific to bacterial membranes, through preferential surface interactions and binding to the anionic headgroups. Nevertheless, the structure and chemical nature of the hydrophobic, helical segment is also an important predictor of antibacterial efficacy, as has been discussed in numerous studies of helical AMPs. Here, for instance, TP4-noR5 shows a lower MIC than NP. This can be interpreted as being due to the more hydrophobic and amphipathic TP4-noR5 helix (compared to the NP), which could promote a stronger helix anchoring between lipid headgroups and insertion into the hydrocarbon region and, thus, cause more profound and irreversible damage to the membrane. This has also been observed with neutron reflectometry and discussed in detail, in our previous studies of P1.^90^ Overall, TP4 is a remarkable example of how two different segments of an AMP play separate yet synergistic roles at bacterial membranes, to achieve an unmatched microbial killing ability.

### Specific Role of Arginine Residues in AMPs’ Structure–Function Relationship

While the total positive charge of AMPs has been acknowledged as an important parameter in membrane activity,^36^ the present study emphasizes the roles that segments of clustered arginines can hold in natural AMPs. We have seen that for TP4, NP-R5 and P1-R5, the polycationic segment has important functions for recruitment to membranes and membrane restructuring through lipid segregation with the consequence of increasing bioactivity. These observations are augmented by our statistical analysis of natural AMP sequences carrying concatenated cationic residues (including lysines) and the reported MIC values for various bacterial species. Interestingly, we found that, clustered R’s positioned either toward the middle or at the C-terminal end of natural AMP sequences, improve the antimicrobial efficacy more than clustered K’s positioned similarly in the peptide chains (Tables 1–3). The same trend is not immediately apparent from the gathered data, if the clustered charge segment in at the N-terminus. This could be due to the lack of catalogued sequences with those characteristics. Indeed, a few isolated examples of highly potent AMPs from all kingdoms of life display clustered R’s at the N-terminus or middle of the sequence, and show MIC values significantly lower than the average AMP. Examples include Myticalins from mollusks,^12^ hLF(1–11) from humans,^15^ protegrin-1 from pigs^16^ and EcAMP3 from grass seed.^21^ Furthermore, the statistical analysis also shows that sequences with clustered R’s are more effective at inhibiting bacteria (lower MIC values) than those with clustered K’s of equivalent charge density, independent of the position of the charges (Figure 2). What molecular features could be responsible for the improvements in bioactivity of clustered R’s versus K’s? A striking feature of arginine is its side-chain, guanidinium ion, with its unique bidentate structure and hydration properties.^109,110^ Neutron diffraction was used to show that the protonated planar guanidinium ion is poorly hydrated (i.e., hydrophobic) above and below the molecular plane while retaining in-plane hydration.^110^ This enables it to easily shed those water molecules and translocate through hydrophobic gaps - a property important for the movement of voltage-sensing segments of ion channels in bilayers^110,111^ and, probably, equally important for AMPs and CPPs. By comparison, the amino group of the side chain of lysine maintains a spherical hydration structure,^112^ impeding translocation. Indeed, it was shown that poly-R peptides can enter cells, while poly-K cannot.^92^ Furthermore, through synchrotron X-ray diffraction studies, peptides with clustered poly arginines, such as TAT peptides, were shown to generate “saddle-shaped” deformations,^113^ enabling a richer structural polymorphism in lipids (and thus, more opportunities to form pores), compared to poly lysines which only generates negative mean curvature leading to “cylinder-shaped” deformations.^92^

Based on all the above, we can surmise that clustered poly-R segments, owing to the unique structural and chemical properties of the guanidium ion, can greatly improve the antibacterial profiles of AMPs by efficient recruitment at bacterial membranes, followed by membrane destabilization through lipid clustering, water retention and intricate local deformations. Notably, the R5 segment is a macro-cation that carries a significant amount of water with it as it penetrates the various layers, opening channels of access, displacing small monovalent and divalent cations and disrupting both the LPS stability and the homeostatic equilibrium of bacteria.^114^ It is likely that the terminal poly-R segment exposed to the membrane surface is targeted for degradation by enzymes like trypsin. The resulting separate fragments could still work independently by releasing a shorter, more hydrophobic segment for faster translocation through the membrane.

## Conclusions

Clustered Arginine (poly-R) segments found in natural and synthetic AMPs are shown here to significantly boost the antimicrobial efficacies of AMPs against both Gram-positive and Gram-negative bacteria. This enhancement in bioactivity can be traced back to preferential interactions of the poly-R motifs of AMPs with lipid components commonly found in bacterial membranes, such as LPS and PG lipids. These favored interactions cause lipid segregation and membrane destabilizations, as revealed by X-ray diffraction. The lipid segregation in domains caused by poly-R segments creates line defects that facilitate anchoring of hydrophobic segments into the hydrocarbon region, thus assisting peptide insertion and translocation. Through database searches and analyses, we show that the presence of poly-R motifs in natural sequences reduces, on average, the minimum inhibitory concentration of AMPs relative to sequences with equivalent charge density but sparsely distributed charged residues, as well as sequences containing clustered poly lysine motifs. The rather limited collection of cataloged natural AMP sequences with recorded bioactivity and biophysical properties makes the poly-R motif rather elusive compared to other, more commonly discussed structural features of AMPs. Finding and harnessing the advantages brought by such impactful sequence motifs circumvents extensive efforts in developing and testing peptide libraries for antibiotic discoveries. TP4 is a remarkable example of how various segments or sequence motifs present in one AMP play separate roles and act synergistically at membranes to achieve unmatched bactericidal effects.

## Supplementary Material



## Notes

Databases; data statistics; MICs and the counts of unique peptide sequences; structure factors for Bragg diffraction peaks; O.D. for 96-well plates; plot of O.D. vs peptide concentration for MIC determination in *V. cholerae* ; DSC scans for LPS and LPS/POPE; XRD data for different lipid systems; neutron reflectometry data; and component volume occupancy profile of TP4 (PDF)
