# Supplementary material for: Poly-Arginine Tails and Helical Segments of Natural Antimicrobial Peptides Display Concerted Action at Membranes for Enhanced Antimicrobial Effects
Source: ACS Bio Med Chem Au. 2025 Jul 8;5(4):706–25. doi: 10.1021/acsbiomedchemau.5c00084 (PMC12371492; doi:10.1021/acsbiomedchemau.5c00084)
Supplement: Supplementary file 1 [file bg5c00084_si_001.pdf]

Supporting information

Poly-Arginine Tails and Helical Segments of Natural Antimicrobial Peptides Display Concerted Action at Membranes for Enhanced Antimicrobial Effects

Navleen Kaur[1], Kinjal Mondal[2,1], Megan E. Mitchell[3], Sarala Padi[4], Jeffery B. Klauda[2,5], Antonio Cardone[4], Frank Heinrich[6,3], Christina R. Harris[7], David K. Giles[7], Mary T. Rooney[8], Erik B. Watkins[9], Myriam L. Cotten[8,10], David P. Hoogerheide[3], Mihaela Mihailescu[1,*]

[1]Institute for Bioscience and Biotechnology Research, Rockville, MD 20850, United States

[2]Institute for Physical Science and Technology, Biophysics Program, University of Maryland, College Park, MD 20742, United States

[3] Center for Neutron Research, National Institute of Standards and Technology, Gaithersburg, MD 20899, United States

[4]Information Technology Laboratory, National Institute of Standards and Technology, Gaithersburg, MD 20899, United States

[5]Department of Chemical and Biomolecular Engineering, University of Maryland, College Park, MD 20742, United States

[6]Department of Physics, Carnegie Mellon University, Pittsburgh, PA 15213, United States

[7] Department of Biology, Geology, and Environmental Science, The University of Tennessee at Chattanooga, Chattanooga, TN 37403, United States

[8]Department of Applied Science, William & Mary, Williamsburg, VA 23185, United States

[9]Oak Ridge National Laboratory, Oak Ridge, TN 37830, United States

[10]Department of Biochemistry and Biophysics, Oregon State University, Corvallis, OR 97331, United States

[*]Corresponding author: E-mail: ella11@umd.edu; Fax: 240-314-6225

TABLE S1: Databases with links to their online web interfaces/sources

Database Source	Link
**GRAMPA**	https://github.com/zswitten/Antimicrobial-Peptides/blob/master/data/grampa.csv
**APD**	https://aps.unmc.edu/
**DBAASP**	https://dbaasp.org/home
**YADAMP**	https://webs.iiitd.edu.in/raghava/satpdb/catalogs/yadamp/
**DRAMP**	https://dramp.cpubioinfor.org/
**StarPep**	https://academic.oup.com/bioinformatics/article/35/22/4739/5474901
**DBAASP3**	https://academic.oup.com/bioinformatics/article/35/22/4739/5474901

TABLE S2. Data statistics of both naturally occurring and synthetic peptide sequences. We have curated 7812 sequences in total, out of which 2486 are natural sequences and 5326 are synthetic sequences. Bacteria Averaged: the average of the other bacteria MIC values.

**Target**	**Curated Sequences**	
	**Natural**	**Synthetic**
E. Coli	1045	4043
S. Epidermidis	147	999
Bacteria Averaged	1294	6518

Table S3. The mean and standard errors of Minimum Inhibitory Concentrations (MIC), in µmol/L, for synthetic peptide sequences, averaged over various bacteria species. (Count) is the number of unique sequences that contain the specified R/K motif. Note: n.a. indicates that there are no sequences with the specified motif.

Motif Size (n)	Position of (n) consecutive arginine/lysine amino acids (**Bacteria Averaged**)					
	START Mean ± Error (Count)		MIDDLE Mean ± Error (Count)		END Mean ± Error (Count)	
	**R**	**K**	**R**	**K**	**R**	**K**
1	20.21±0.48 (2214)	22.53±0.38 (3974)	20.21± 0.46 (2513)	22.09± 0.36 (4261)	21.70±0.44 (2958)	23.48± 0.36 (4615)
2	20.12±1.14 (431)	17.65±0.60 (1216)	17.93± 1.00 (550)	21.22± 0.74 (873)	20.15± 0.95 (587)	20.51± 0.64 (1189)
3	21.93±3.34 (41)	20.08± 2.15 (71)	22.19± 3.66 (57)	18.68± 3.36 (43)	19.65± 2.49 (76)	18.70± 1.82 (98)
4	18.55±4.88 (11)	14.89± 3.11 (20)	27.64 ± 11.88 (6)	11.15±3.82 (10)	31.20± 5.98 (17)	22.11± 4.24 (18)
5	17.14±5.38 (7)	15.46± 3.71(16)	47.83±16.59 (3)	12.29±9.39 (2)	32.68± 8.39 (9)	24.82±22.04 (2)
6	23.33±7.91 (4)	5.43± 2.54(4)	n.a.	n.a.	39.22± 11.80 (3)	n.a.

Table S4. The mean and standard error of MIC values, in μmol/L, for synthetic peptide sequence, for ***E. coli.*** (Count) is number of sequences that contain the specified R/K motif. Note: n.a. indicates that there are no sequences with the specified motif.

Motif Size (n)	Position of (n) consecutive arginine/lysine amino acids (***E. coli***)					
	START Mean ± Error (Count)		MIDDLE Mean ± Error (Count)		END Mean ± Error (Count)	
	**R**	**K**	**R**	**K**	**R**	**K**
1	18.86±0.61 (1685)	19.55±0.47 (2996)	19.23± 0.58 (1934)	20.08± 0.45 (3234)	20.47± 0.55 (2204)	21.60± 0.45 (3436)
2	18.53±1.35 (343)	14.27±0.71 (920)	16.66± 1.24 (396)	18.85± 0.96 (635)	17.89± 1.11 (418)	19.72± 0.83 (860)
3	12.31±3.78 (29)	17.27± 4.65 (26)	8.91± 1.99 (33)	25.9165± 6.87 (24)	12.967± 2.76 (46)	19.53± 3.07 (61)
4	9.89±5.78 (8)	7.76± 3.50 (9)	7.54 ± 2.42 (3)	18.03±9.72 (4)	14.04± 4.19 (10)	28.50± 6.18 (19)
5	2.65±0.80 (4)	4.76± 1.78(7)	n.a.	16.99±14.50754 (2)	12.1± 4.15 (6)	9.58±2.91(2)

Table S5. The mean and standard error of MIC values, in µmol/L, for synthetic peptide sequences, for ***S. epidermidis***. (Count) is number of sequences that contain the specified R/K motif. Note: n.a. indicates that there are no sequences with the specified motif.

Motif Size (n)	Position of (n) consecutive arginine/lysine amino acids (***S. epidermidis***)					
	START Mean ± Error (Count)		MIDDLE Mean ± Error (Count)		END Mean ± Error (Count)	
	**R**	**K**	**R**	**K**	**R**	**K**
1	11.94±0.96 (360)	14.30±0.94 (520)	14.62± 1.29 (299)	13.53± 0.78 (630)	14.85± 1.10 (399)	14.78± 0.84 (660)
2	11.81±2.19 (79)	12.90±1.55 (164)	11.56± 2.80 (45)	13.00± 1.43 (178)	11.90± 1.81 (90)	14.44± 1.72 (187)
3	8.46±1.54 (7)	9.57± 3.61 (8)	13.84± 8.58 (7)	4.61± 1.31 (8)	8.65± 2.75 (10)	20.68± 5.35 (21)
4	n.a.	5.42± 1.06 (5)	n.a.	6.75±5.75 (2)	1.66± 0.33 (3)	25.55± 7.73 (13)

(A)

	1	2	3	4	5	6	7	8	9	10	11	12		
A	0.187	0.126	0.13	0.102	1.036	1.026	1.075	1.138	1.564	1.168	1.415	0.102	600	TP4
B	0.156	0.166	0.098	0.102	1.354	1.054	1.331	1.208	1.047	1.163	1.445	0.099	600	TP4
C	0.161	0.145	0.127	0.096	1.076	1.107	1.272	1.167	1.221	1.395	1.552	0.108	600	TP4
D	0.104	0.093	1.756	0.966	0.893	0.907	0.838	0.732	1.668	1.636	1.577	0.114	600	TP4-noR5
E	0.127	0.09	0.883	1.429	0.828	1.057	0.54	0.609	1.465	1.676	1.638	0.119	600	TP4-noR5
F	0.112	0.103	0.797	1.303	0.856	0.677	1.181	1.432	1.617	1.668	1.659	0.112	600	TP4-noR5
G	0.094	0.091	0.092	0.093	0.091	0.094	0.094	0.092	0.098	0.095	0.094	0.093	600	
H	0.098	0.097	0.092	0.091	0.093	0.102	0.104	0.091	0.095	0.094	0.093	0.099	600	
	32 µM	16 µM	8 µM	4 µM	2 µM	1 µM	0.5 µM	0.25 µM	0.125 µM	0.0625 µM	Bacteria	Medium		

(B)

	1	2	3	4	5	6	7	8	9	10	11	12		
A	0.196	0.123	0.107	0.101	0.104	0.101	0.948	0.767	0.652	0.675	0.959	0.49	600	TP4
B	0.432	0.334	0.111	0.1	0.098	0.104	1.01	0.665	0.978	0.889	1.084	0.103	600	TP4
C	0.289	0.174	0.145	0.099	0.098	0.1	1.198	0.702	0.721	0.818	0.97	0.095	600	TP4
D	0.267	0.104	0.1	0.108	0.112	1.337	1.044	1.034	1.081	0.838	1.038	0.096	600	Tp4-noR5
E	0.185	0.1	0.099	0.136	0.28	1.416	1.004	0.805	0.97	0.877	0.953	0.093	600	Tp4-noR5
F	0.231	0.228	0.114	0.115	0.256	1.063	0.964	1.529	0.89	0.836	0.919	0.116	600	Tp4-noR5
G	0.106	0.095	0.094	0.095	0.095	0.103	0.096	0.096	0.095	0.094	0.094	0.099	600	
H	0.105	0.096	0.098	0.098	0.094	0.101	0.103	0.091	0.095	0.116	0.097	0.099	600	
	32 µM	16 µM	8 µM	4 µM	2 µM	1 µM	0.5 µM	0.25 µM	0.125 µM	0.0625 µM	Bacteria	Medium		

Figure S1. Bacterial susceptibility assay. Optical density of each well of a 96-well plate determined at 600 nm using a BioTek plate reader after incubation of (**A**) *E. coli* and (**B**) *S. epidermidis* with different concentrations of peptides TP4 and TP4-noR5 for 24 h at 37 °C. Last row mentions the concentration of peptides in each well. Columns tested for positive and negative blanks are mentioned as Bacteria and Medium.

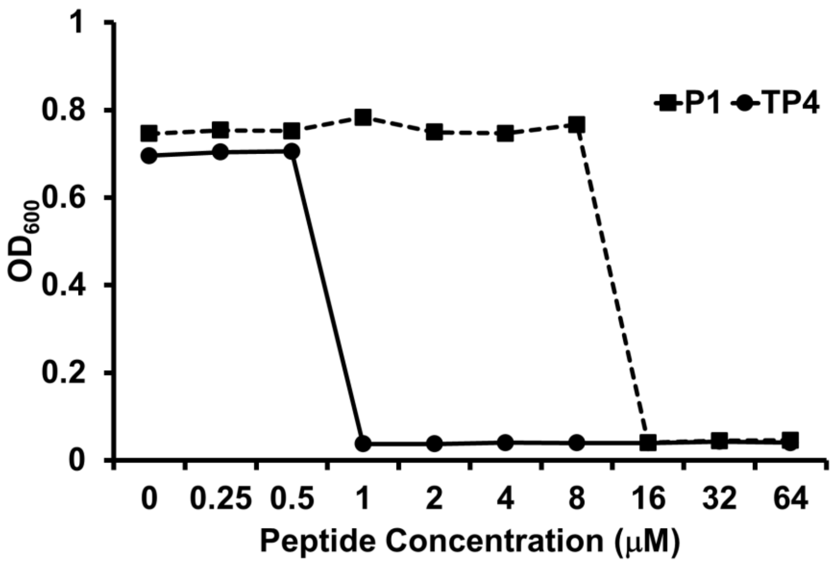

Figure S2. MIC Determination of P1 and TP4 in *V. cholerae*. Bacteria were grown at 37°C in CM9-HEPES (pH 7.4) to mid-log phase (OD_{600} = 0.8-0.9). Cultures were pelleted, washed with media and resuspended. The bacterial suspension was distributed into microtiter plates and two-fold concentrations of P1 and TP4 were added. After 20 h incubation, the optical density (600 nm) was read using a Biotek Synergy microplate reader. Experiments were conducted in triplicates, with each value representing the mean (all standard deviations < 0.03). Two independent assays were done in triplicates. One representative assay is displayed here. MIC of P1 = 16 μmol/L; MIC of TP4 = 1 μmol/L. Bacterial death at the determined MIC was verified by plating of sample wells on Luria agar.

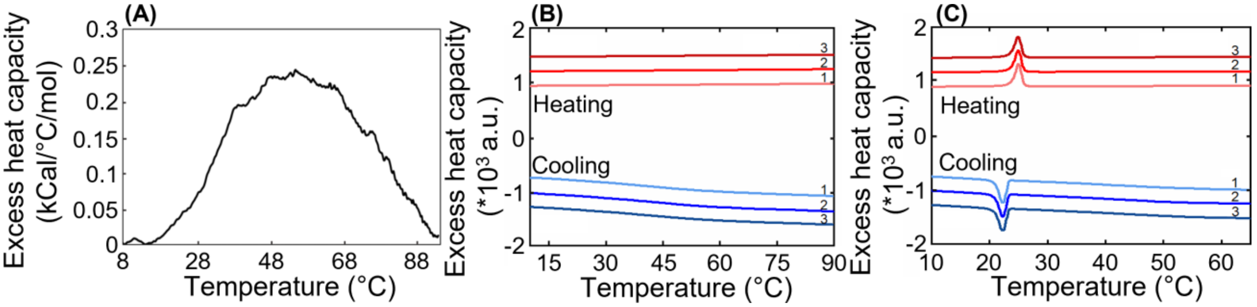

Figure S3. Differential Scanning Calorimetry (DSC) data of lipid SUVs containing lipopolysaccharide (LPS). (**A**) DSC heating scan for LPS at a concentration of 1.25 mg/mL in water. (**B**) and (**C**) Raw DSC scans. DSC heating and cooling consecutive scans for (**B**) LPS (1.25 mg/mL), (**C**) LPS/POPE (molar ratio 1:5) (2.08 mmol/L). All samples were prepared in water. Scan rate was 0.5 ^{o}C/min.

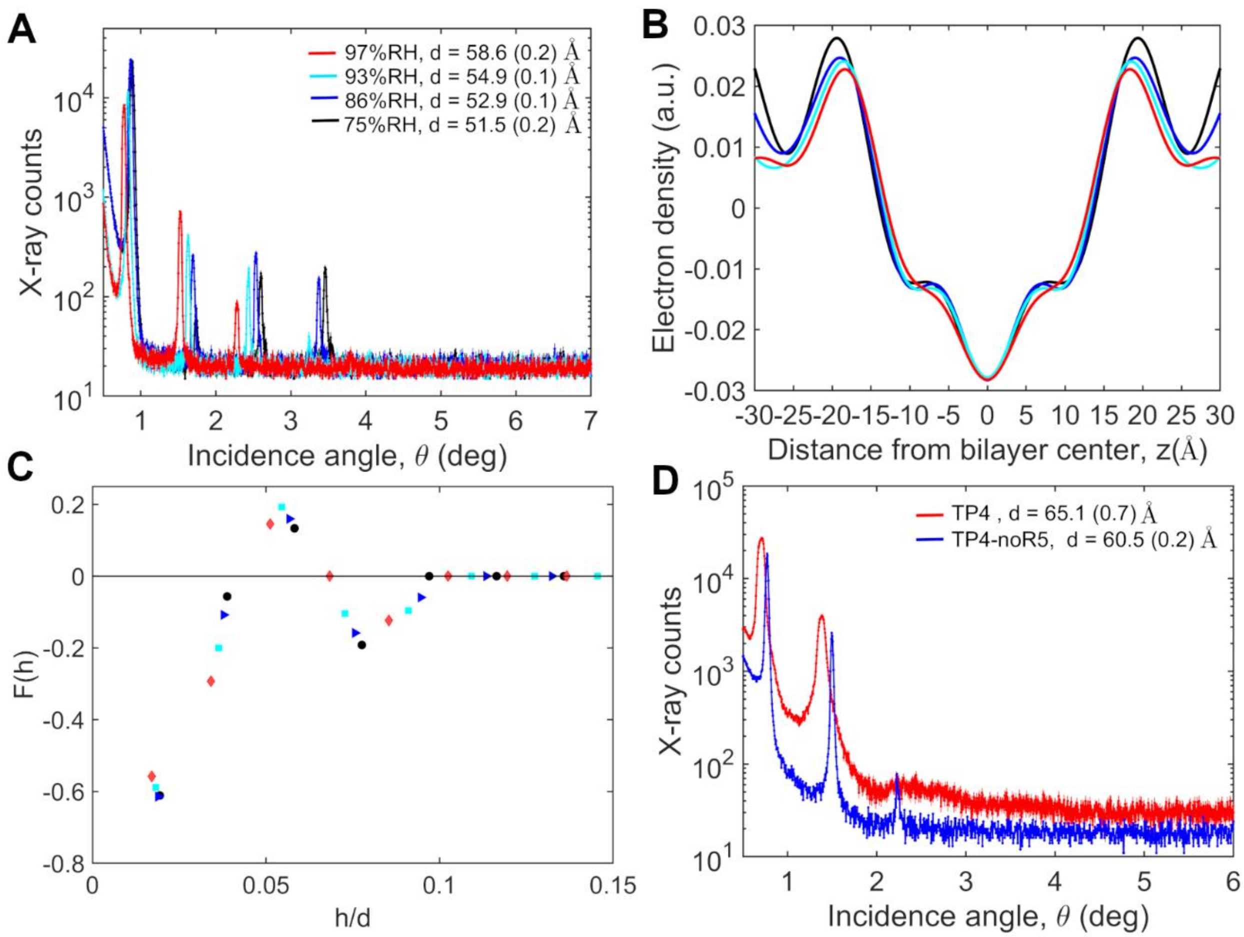

Figure S4. X-ray diffraction data from oriented multilayer samples of LPS/POPC. (**A**) Diffraction patterns from LPS/POPC (1:5 molar ratio) lamellar samples at various hydrations attained with saturated salt solution, at 25 ^{o}C. (**B**) Electron density profiles corresponding to the data in (A). **(C)** The variation in the structure factor F(h) with the reciprocal spacing h/d, corresponding to the data in A, for determining the phases (+ or -) of the structure factors for the centro-symmetric bilayer system. h is the diffraction (Mueller) index and d is the repeat spacing. (**D**) Diffraction pattern for TP4-noR5 and TP4 in LPS/POPC (1:5), for a P/L = 1/50, from lamellar samples measured at 97% RH and 25 ^{o}C. Note the significant broadening and repeat spacing increase in the presence of TP4, compared to TP4-noR5.

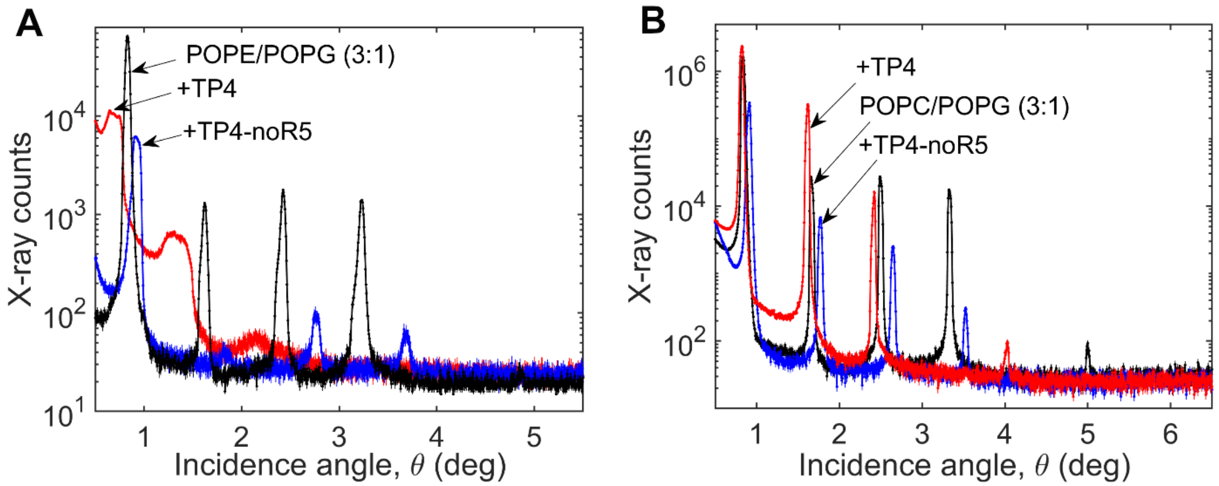

Figure S5. X-ray diffraction data from oriented multilayer samples of POPE/POPG and POPC/POPG. (**A**) Diffraction patterns for POPE/POPG (3:1 molar ratio) (black), and in the presence of + TP4 (red) and TP4-noR5 (blue). Samples were at a P/L of 1:25, measured at 97% RH and 25 °C. Note the pronounced phase separation caused by TP4. (**B**) Diffraction patterns for POPC/POPG (3:1 molar ratio) (black), and in the presence of TP4 (red) and TP4-noR5 (blue). Samples were at a P/L of 1:25, measured at 93% RH and 25 °C.

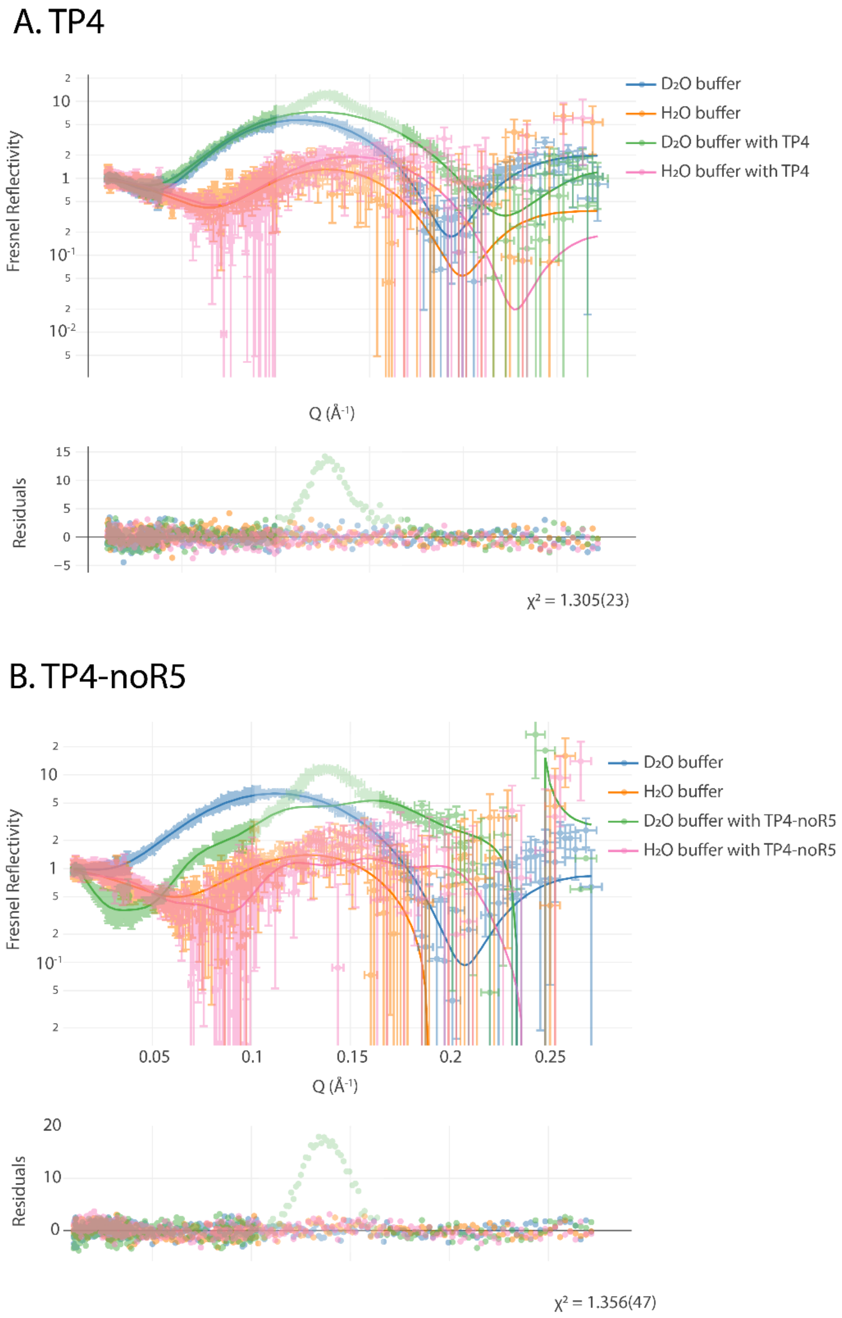

Figure S6. Neutron reflectometry (NR) data for bilayers with AMPs. NR for 3DOPE:1POPG lipid bilayers adsorbed on silicon wafers and subsequently exposed to (**A**) TP4 or (**B**) TP4-noR5 dissolved at 3 µmol/L in 10 mmol/L tris buffered at pH 7.4. Where specified, buffers also contain 150 mmol/L NaCl. Data are normalized to the Fresnel reflectivity of the silicon/buffer interface. Uncertainties represent 68% confidence intervals derived from the neutron counting uncertainties. Curves are reflectivity patterns optimized to the models described in the main text. The multilayer peaks were removed from the analysis (shading), and the optimized reflectivity patterns were obtained without these data. Goodness of fit metrics were (A) $\chi^2 = 1.305$ and (B) $\chi^2 = 1.356$.

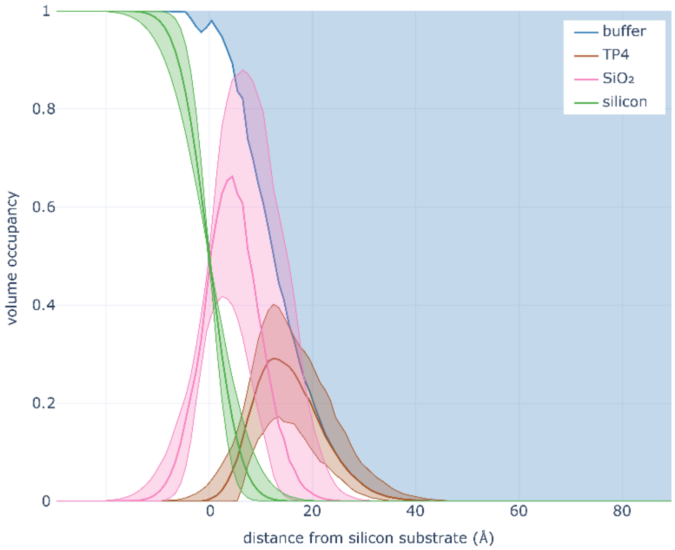

Figure S7. Component volume occupancy profile of TP4 adsorbed to a Si/SiO_2 interface in the absence of lipids. The total volume of peptide is consistent with a complete layer $5.4^{+1.5}_{-2.5}$ Å thick, or about 60% of an 8 Å peptide monolayer.

TABLE S6. Structure factors F(h) and standard deviation (S.D.) of all observed Bragg diffraction peaks (n) for oriented LPS-Re/POPC (1:5) bilayers at various relative humidities of 75%-97%. The values correspond to data in **Figure S4A**. Samples were made in H_2O and measured at 25 °C.

	75% RH *d=51.5 ± 0.2		86% RH d=52.9 ± 0.1		93% RH d=54.9 ± 0.1		97% RH d=58.6 ± 0.1	
**h**	**F(h)**	**S.D.**	**F(h)**	**S.D.**	**F(h)**	**S.D.**	**F(h)**	**S.D.**
1	0.611	0.001	0.614	0.001	0.588	0.001	0.557	0.001
2	0.057	0.003	0.108	0.002	0.200	0.002	0.293	0.002
3	0.133	0.003	0.160	0.003	0.192	0.004	0.145	0.006
4	0.192	0.005	0.158	0.006	0.105	0.012	0.107	0.010
5	0.055	0.020	0.059	0.012	0.096	0.012	0.123	0.016
6	0.084	0.026	0.063	0.018	0.122	0.011	0.171	0.014
7	0.066	0.040	0.075	0.028	0.108	0.033	0.128	0.029
8	0.075	0.036	0.101	0.030	0.108	0.025	0.151	0.031

* d - the repeat spacing in units of Angstrom

TABLE S7. Structure factors F(h) and standard deviation (S.D.) of all observed Bragg diffraction peaks, of index (h), for oriented POPC and LPS/POPC (1:5) bilayers and in the presence of TP4 and TP4-noR5 at a relative humidity of 93%. The values correspond to data in **Figures 8C** and **D** in the main manuscript. Samples were made in H_2O and measured at 25 °C.

	**POPC** *d=52.6 ±0 .1		**LPS-Re/POPC** d=55.5 ± 0.1		**TP4 in LPS-Re/POPC P/L=1:50** d=56.2 ± 0.3		**TP4-noR5 LPS/POPC P/L=1:50** d=55.0 ± 0.1		**TP4-noR5-LPS/POPC P/L=1:25** d=55.0 ± 0.1	
**h**	**F(h)**	**S.D.**	**F(h)**	**S.D.**	**F(h)**	**S.D.**	**F(h)**	**S.D.**	**F(h)**	**S.D.**
1	-2.527	0.001	-2.505	0.001	-2.631	0.001	-0.977	0.001	-2.393	0.001
2	-0.634	0.001	-1.004	0.001	-0.625	0.001	-0.459	0.002	-1.057	0.004
3	0.920	0.002	0.922	0.003	0.229	0.003	0.286	0.004	0.273	0.009
4	-1.034	0.002	-0.503	0.006	0.000	0.017	-0.084	0.014	-0.097	0.038
5	0.070	0.018	-0.304	0.028	-0.107	0.017	-0.090	0.025	0.000	0.041
6	-0.163	0.012	0.000	0.043	0.000	0.029	0.000	0.020	0.000	0.064
7	0.000	0.039	0.000	0.031	0.000	0.032	0.000	0.050	#n/m	
8	0.000	0.070	0.000	0.038	0.000	0.024	0.000	0.030	n/m	

*d - the repeat spacing in units of Angstrom. #n/m - not measured.

TABLE S8. Structure factors F(h) and standard deviation (S.D.) of all observed Bragg diffraction peaks (n) for oriented POPE/POPG (3:1) bilayers at relative humidity of 97%. The values correspond to data in **Figure 9A**. Samples were made in H_2O and measured at 30 °C.

	**POPE/POPG (3:1)** *d=51.7 ± 0.1		**NP-POPE/POPG (3:1) P/L=1:25** d=55.1 ± 0.1		**TP4-noR5-POPE/POPG (3:1) P/L=1:25** d=47.2 ± 0.03	
**h**	**F(h)**	**S.D.**	**F(h)**	**S.D.**	**F(h)**	**S.D.**
1	-5.281	0.001	-7.312	0.002	-6.792	0.009
2	-0.383	0.002	-0.163	0.011	-0.000	0.093
3	0.872	0.002	0.714	0.008	1.843	0.094
4	-2.482	0.002	-1.068	0.008	-2.191	0.090
5	0.441	0.007	-0.245	0.039	0.000	0.308
6	-0.534	0.006	-0.225	0.072	0.000	0.852
7	-0.172	0.024	0.000	0.142	0.000	0.326
8	-0.303	0.023	0.000	0.126	0.000	0.355

* d - the repeat spacing in units of Angstrom
